# HD‐ZIP IV gene *Roc8* regulates the size of bulliform cells and lignin content in rice

**DOI:** 10.1111/pbi.13435

**Published:** 2020-07-08

**Authors:** Jing Sun, Xuean Cui, Shouzhen Teng, Zhao Kunnong, Yanwei Wang, Zhenhua Chen, Xuehui Sun, Jinxia Wu, Pengfei Ai, William Paul Quick, Tiegang Lu, Zhiguo Zhang

**Affiliations:** ^1^ Joint CAAS/IRRI Laboratory for Photosynthetic Enhancement Biotechnology Research Institute/National Key Facility for Genetic Resources and Gene Improvement The Chinese Academy of Agricultural Sciences Beijing China; ^2^ College of Bioscience and Bioengineering Hebei University of Science and Technology Hebei China; ^3^ C4 Rice Center International Rice Research Institute (IRRI) UPLB Los Baños Laguna Philippines; ^4^ Department of Animal and Plant Sciences University of Sheffield Western Bank Sheffield UK

**Keywords:** rice, *Roc8*, 3′‐UTR, bulliform cells, lignin, photosynthesis

## Abstract

The morphology of bulliform cells located on the upper epidermis of leaves is one of the most important cell structures affecting leaf shape. Although many mechanisms regulating the development of bulliform cells have been reported, the fine regulatory mechanisms governing this process have rarely been described. To identify novel components regulating rice leaf morphology, a mutant showing a constitutively rolling phenotype from the seedling stage to flowering, known as *crm1‐D*, was selected for further analysis. Anatomical analyses in *crm1‐D* were attributable to the size reduction of bulliform cells. The *crm1‐D* was controlled by a single dominant nuclear gene. Map‐based cloning revealed that *Roc8,* an HD zipper class IV family member, was responsible for the *crm1‐D* phenotype. Notably, the 50‐bp sequence in the 3′‐untranslated region (3′‐UTR) of the *Roc8* gene represses *Roc8* at the translational level. Moreover, the *roc8* knockdown lines notably increased the size of bulliform cells. A series of assays revealed that *Roc8* negatively regulates the size of bulliform cells. Unexpectedly, *Roc8* was also observed to positively mediate lignin biosynthesis without incurring a production penalty. The above results show that *Roc8* may have a practical application in cultivating materials with high photosynthetic efficiency and low lignin content.

## Introduction

Leaf morphology is an important part of the ideal plant architecture of rice (Zhang *et al*., 2009). Moderate leaf curling helps to keep the leaves upright, increase the light‐receiving space at the base of the middle and late stages of population, improve the utilization rate of light energy and enhance the light quantity and intensity at the base of the canopy (Xu *et al*., 2018). Moderate leaf curling also helps to improve root system activity and strengthen lodging resistance (Zhang *et al*., 2009; Zou *et al*., 2011). Therefore, the study of leaf morphology has become the focus of basic theory and practical breeding applications.

Considerable progress has been made in genetic studies of leaf morphogenesis in rice (Xu *et al*., 2018). Leaf curling was primarily caused by bulliform cell changes in monocotyledons. Bulliform cells, also called motor cells, are among the most important cell structures and are located on the upper epidermis of leaves. The osmotic pressure change of bulliform cells leads to leaf curling. Physiological studies showed that under water stress, bulliform cells shrank, and the leaves rolled. When the stress was removed, the bulliform cells swelled and absorbed water, and the leaves returned to their original state (Sylvester *et al*., 2001; Zou *et al*., 2011). To date, at least 10 genes controlling bulliform cell development have been reported in rice (Xu *et al*., 2018). These genes mainly included *BRD1* (Hong *et al*., 2002), *NRL1* (Hu *et al*., 2010), *RL14* (Fang *et al*., 2012), *SRL1* (Xiang *et al*., 2012), *LC2* (Zhao *et al*., 2010), *Roc5* (Zou *et al*., 2011), *ACL1/ACL2* (Li *et al*., 2010) and *OsLBD3‐7* (Li *et al*., 2016). These genes played an important role in controlling the number and size of bulliform cells*. NRL1, RL14* and *ACL1/ACL2* positively regulated the development of bulliform cells, while *SRL1, BRD1, LC2, Roc5* and *OsLBD3‐7* negatively regulated the development of bulliform cells. Most of the genes encoded oxidative, reductive or synthetase and were located at the lowest level of the signalling pathway. Despite several findings, the molecular mechanism of leaf rolling has not been fully elucidated. Few detailed studies have been conducted on directly regulating the development of bulliform cells by transcription factors.

Notably, the transcription factor Roc5 encodes an HD zipper class IV family member that negatively regulates bulliform cell development in combination with downstream target gene protodermal factor‐like (Zou *et al*., 2011). Whether other *Roc* families in rice have similar mechanism remains to be explored. In this study, a *crm1‐D* mutant showing a rolling phenotype was selected for analysis. Roc8, an HD zipper class IV family member, was responsible for the *crm1*‐D phenotype. We verified that the 50‐bp sequence in the *Roc8* 3′‐UTR represses Roc8 at the translational level. *Roc8* was observed to negatively regulate the size of bulliform cells. In particular, *Roc8* was determined to positively modulate leaf lignin contents in rice leaves. The results of this study may be helpful to understand the function of the rice outermost cell‐specific gene (Roc) family and provide a potential theoretical basis for cultivating materials with high photosynthesis or low lignin contents in rice or forage grass.

## Results

### Phenotypic characterization of the* crm1*‐*D* mutant

**Figure 1:**
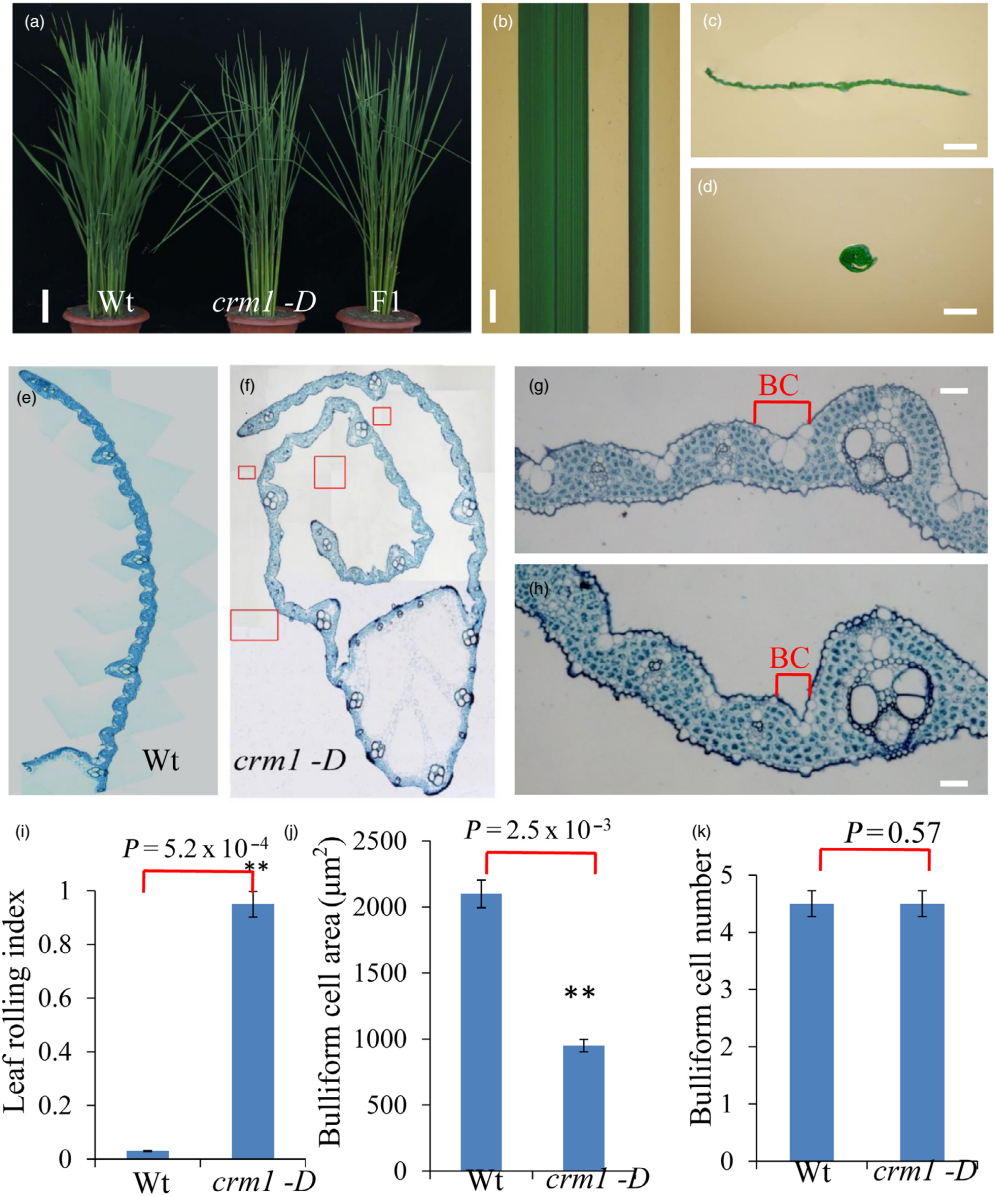
Phenotypic comparison of *crm1‐D* and wild‐type plants. (a) Plant stature of Wt, *crm1‐D* and F1 plants (bar = 5 cm). (b) Leaf phenotypes of Wt (left) and *crm1‐D* mutant (right) at the tiller stage (bar = 1 cm). (c‐d) Transverse leaf sections of Wt (c) and *crm1‐D* mutants (d) at the tiller stage (bar = 0.5 cm). (e) Composite image of mature leaf blade of Wt from midvein to leaf margin. (f) Composite image of the mature leaf blade of the *crm1‐D* mutant. In order to better display the paraffin section results of *crm1‐D*, f is generated by *crm1‐D* multiple field photographs (using the Photoshop6 software overlap function). Red boxes represent the overlapping position of *crm1‐D* photographs with different vision fields. (g) Wild‐type leaf showing the placement of bulliform cells (BCs) between a major and minor vein (bar = 20 μm). (h) *crm1‐D* mutant leaf showing reduced size of bulliform cells (bar = 20 μm). (i‐k): (i) Leaf rolling index (*n* = 10), (j) bulliform cell area and (k) bulliform cell number of wild‐type and mutant plants (*n* = 10). Bars represent the SD of measurements. Asterisks denote significant differences based on Student’s *t*‐test (**P < 0.01).

To identify novel components regulating rice leaf morphology, a mutant named *crm1‐D* showed a constitutively inward rolling phenotype from the seedling to flowering stage (Figure 1a, b, c, d), which was selected for analysis. The rolling degree in *crm1‐D* adds up to ~0.9 compared with the wild type (Figure 1i). F_1_ crosses of *crm1‐D* with the wild type suggested that the recovered allele was dominant (Figure 1a). Genetic analyses of heterozygous F_1_ progeny showed that the phenotype in *crm1‐D* segregated in a 3:1 ratio (*χ* ^2^ = 1.1145, χ^2^ < 0.05, 1) of wild‐type (45) and mutant‐like (156) plants, indicating that the *crm1‐D* phenotype was caused by a single dominant mutation.

A detailed histological examination of the *crm1‐D* mutant leaves identified no gross abnormalities in leaf ultra‐structure with the exception of bulliform cells that appeared smaller in the mutant relative to the wild type (Figure 1e, f). At the mature stage, the number of bulliform cells in *crm1‐D* was equivalent to that in wild type (Figure 1k), but the size of bulliform cells in *crm1‐D* was considerably smaller than that in wild type (Figure 1g, h, j). The reduced size of bulliform cells in *crm1‐D* was correlated with increased stomatal conductance (Figure S1b, Method S17), increased transpiration rates (Figure S1c), reduced photosynthetic capacity (Figure S1a) and higher internal CO_2_ level of the leaf (Figure S1d) relative to the wild type. Collectively, these results suggest that plant morphological changes in *crm1‐D* plants caused by the size of bulliform cells led to a reduction in photosynthetic efficiency.

Bulliform cells can be stained by toluidine blue O (Hernandez *et al*., 1999). The adaxial leaf *crm1‐D* and wild‐type plants at the 10th leaf stage were peeled, and bulliform cells and epidermal cells were stained purple and blue, respectively, with toluidine blue O (Figure S2a). The cross section of seedling leaves of *crm1‐D* and wild‐type plants were observed by scanning electron microscopy (Figure S2b). The size and area of bulliform cells in *crm1‐D* were significantly reduced compared with wild type (Figure S2a, b), again demonstrating decreased size of bulliform cells in *crm1‐D* compared with the wild type.

### Map‐based cloning of *CRM1*

**Figure 2:**
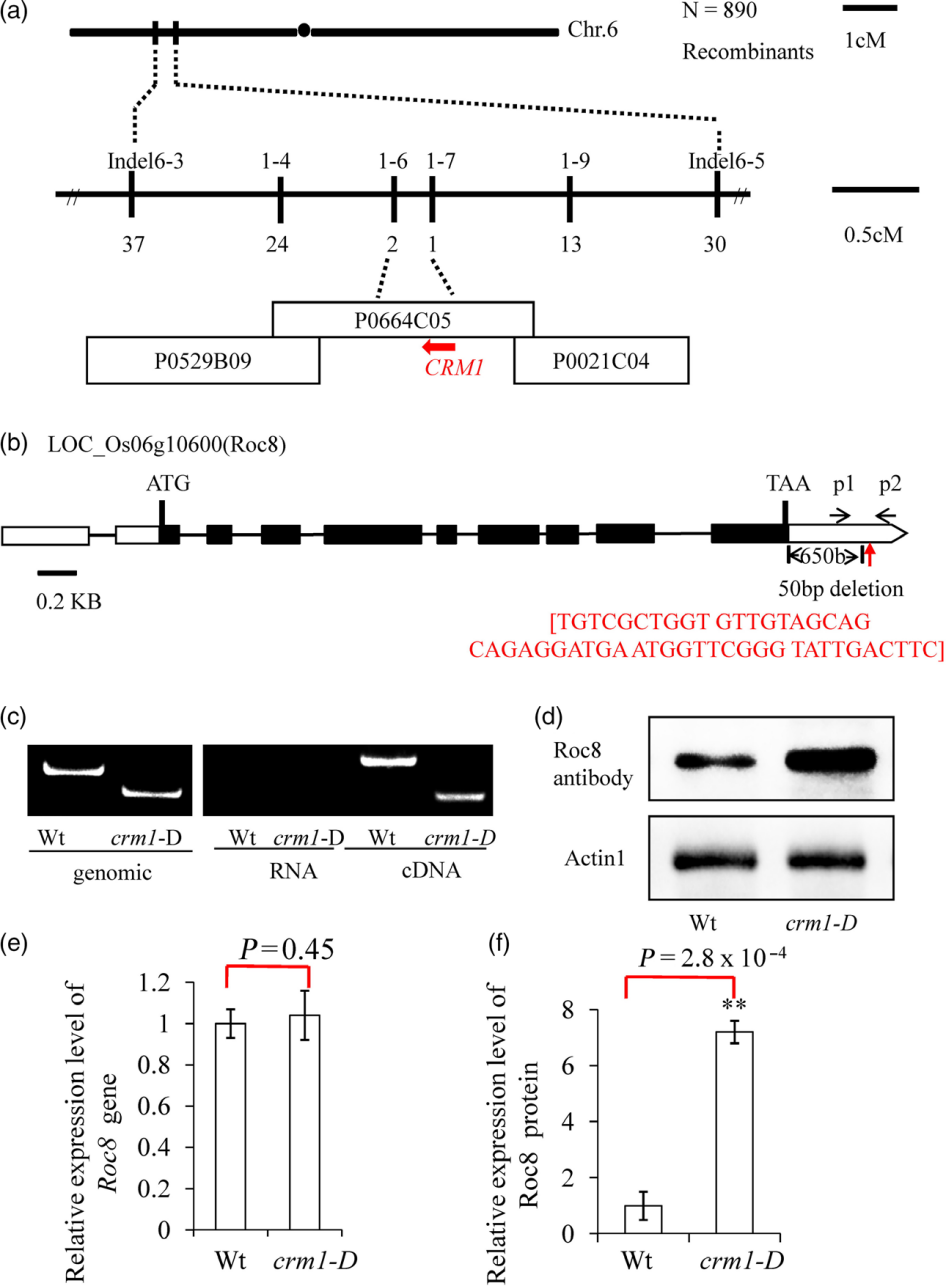
Map‐based cloning of the *CRM1* gene. (a) The CRM1 locus was mapped to a 5‐cM interval on chromosome 6 between Indel markers Indel6‐3 and Indel6‐5. The location of the CRM1 locus was fine‐mapped to a 30‐kb region between InDel markers 1–6 and 1–7 on BAC clone P0664C05 using 890 F2 homozygous plants. There were five open reading frames (ORF1‐ORF5) as potential candidate genes in this interval. (b) The structure of CRM1 (ORF3). Putative ATG start and TAA stop codons are shown. A 50‐nt deletion 658 bp downstream of TAA was detected in *crm1‐D*. (c) P1 and P2 primers shown in (b) were used to distinguish the CRM1 deletion region from wild type and *crm1‐D* in genomic DNA (left). PCR amplification of products from RNA (no bands) and cDNA confirmed the presence of a 50‐nt deletion present in *crm1*‐D transcripts. (d) Western blot analysis was performed using a Roc8 polyclonal antibody generated against the full‐length Roc8 fusion protein. (e) Comparison of transcript abundance of Roc8 in wild‐type (Wt) and *crm1‐D* mutant by qRT–PCR. (f) Comparison of protein abundance of Roc8 in wild‐type (Wt) and *crm1*‐D mutants by quantification of signal from Western blot using ImageJ analysis. Bars represent the SD of measurements. Asterisks denote significant differences based on Student’s *t*‐test (**P < 0.01).

The *crm1‐D* mutant was coarsely mapped in an F_2_ population to the short arm of rice chromosome 6 between markers Indel6‐3 and Indel6‐5 (Figure 2a). To fine‐map the *CRM1*, we screened 890 individuals using markers designed to the region between Indel6‐3 and Indel6‐5 (Figure 2a). The *CRM1* locus was further delimited to a 30‐kb region, which contained five putative open reading frames located between markers Indel1‐6 and Indel1‐7 (Figure 2a).

To identify the candidate gene in this interval, quantitative PCR was used to measure transcript levels of each of the five ORFs (LOC_Os06g10580, LOC_Os06g10590, LOC_Os06g10600, LOC_Os06g10610 and LOC_Os06g10620) in the *crm1‐D* mutant and wild type. Surprisingly, no significant differences in gene expression in any of the genes were detected between wild‐type and *crm1‐D* plants in tissues from the 3rd leaf stage (Figure 2e, Figure S3a, b, c, d and Method S4). However, sequence analysis of the ORFs revealed a 50‐bp deletion located 650 bp downstream of the stop codon of LOC_Os06g10600 (Figure 2b). This gene was predicted to encode *Roc8*, a putative homeodomain leucine zipper class IV gene (Ito *et al*., 2002), closely related to *Roc5*, a gene previously shown to regulate leaf rolling in rice (Zou *et al*., 2011). The *Roc8* gene may be an optimum candidate gene. *Roc8* is predicted to encode a transcript of 3070 nucleotides with an open reading frame of 2094 nucleotides that direct the synthesis of a 697 amino acid protein of predicted molecular mass of 75 kD. The coding region of *Roc8* consists of nine exons interrupted by eight introns (Figure 2b). The Roc8 protein is predicted to encode two conserved domains, a DNA‐binding HD‐ZIP at the N‐terminus and a putative lipid‐interacting START domain at the C‐terminus (Figure S5a). Roc8 shares 76.52% protein identity with the maize homolog OCL1, which is highly expressed in immature tassels, including meiotic tissues and anthers (Depege‐Fargeix *et al*., 2011). Roc8 also shares extensive sequence homology with GL2, previously shown to regulate trichome differentiation in *Arabidopsis thaliana* (Khosla *et al*., 2014).

### Roc8 transcription profiling

Data submitted to the microarray transcript profiling database indicated that *Roc8* was widely expressed in rice with strong expression in leaf tissue (Zimmermann *et al*., 2004). To validate these data, we conducted an expression analysis by qRT‐PCR using RNA samples prepared from wild‐type tissues at the seedling and mature stages. *Roc8* transcript abundance was highest in young leaf blade and sheath tissues and lowest in root and culm tissues (Figure S4).

**Figure 3:**
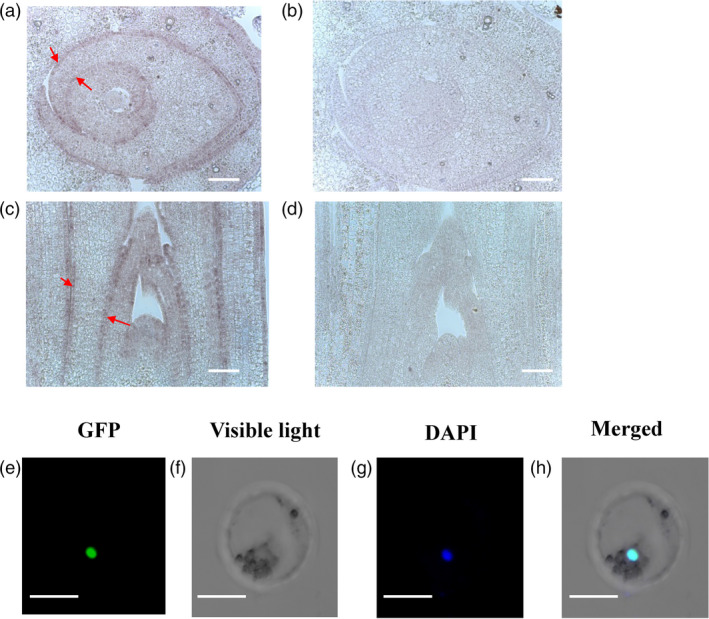
*In situ* hybridization and subcellular localization of Roc8. (a‐d) *In situ* hybridization using fragments from the partial coding sequence of the Roc8 gene. (a) Transverse or (c) longitudinal sections through the shoot apex of six‐day‐old seedlings were hybridized with a digoxigenin‐labelled (a, c) antisense probe or (b, d) sense strand probe of Roc8. Arrows denote both adaxial and abaxial expression of Roc8 restricted to the L1 layer of developing leaves. c, d: bar = 100 μm. a, b: bar = 20 μm. (e)–(h) A 35S::Roc8:GFP vector was transformed into rice protoplasts, and green fluorescent signals were visualized using confocal microscopy. DAPI staining of nuclei revealed that the GFP signal colocalized with DAPI fluorescence, indicating that Roc8 was exclusively targeted to the rice nucleus. e‐h: bar: 100 µm.

An *in situ* hybridization assay was used to detect the expression pattern of the *Roc8* gene. Shoot apexes of cross section and longitudinal cutting of six‐day seedlings were probed with digoxigenin‐labelled sense or antisense strand of *Roc8* RNA. The results showed that in the sense probe, there were no visible hybridization signals, whereas strong signals were expressed in the epidermal cell and vascular bundle using antisense probe hybridization (Figure 3a, b, c, d). This result indicated that Roc8 may function in epidermal cell differentiation and vascular bundle development.

**Figure 4:**
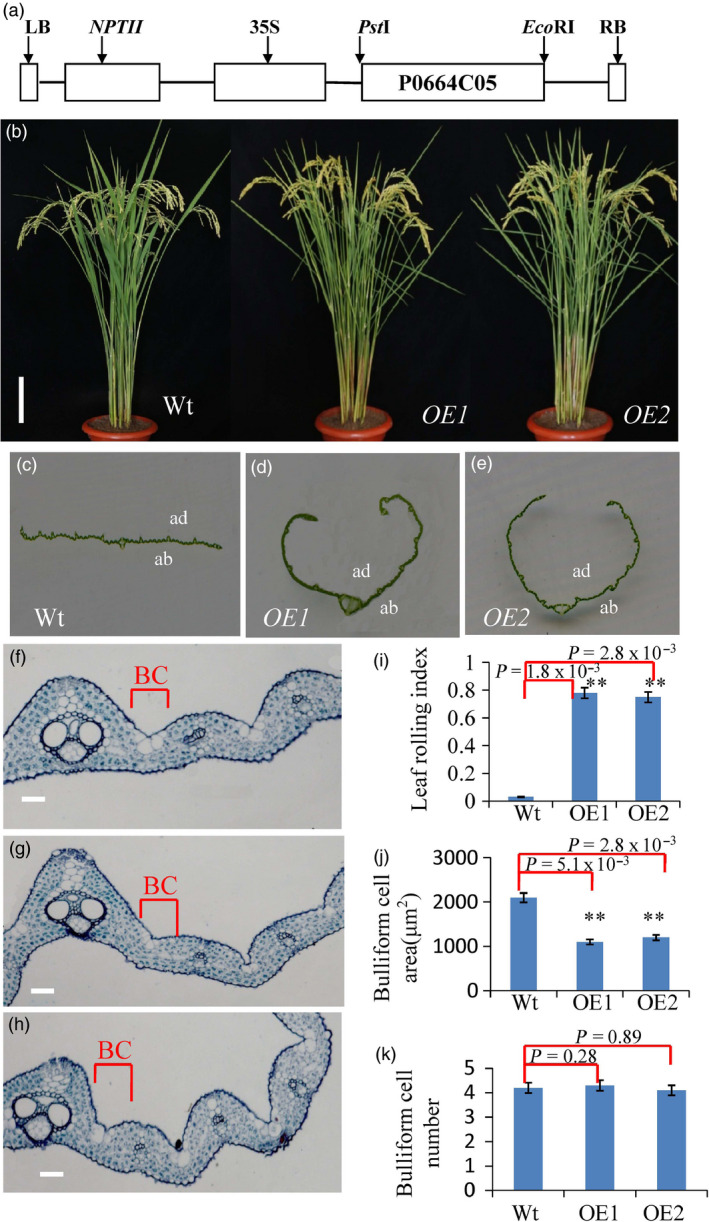
Overexpression of Roc8 recapitulates the *crm1‐D* Phenotype. (a) Schematic of the pCRM1 expression vector containing the full Roc8 open reading frame derived by Pst1/EcoRI digestion of BAC P0664C05. (b) Plant stature of Wt and Roc8 overexpression in T_1_ plants (OE1, OE2) at maturity. (c‐e) Leaf phenotypes of (c) Wt, (d) OE1 and (e) OE2 at maturity (ab: abaxial side; ad: adaxial side, bar = 5 cm). (f) Transverse section of Wt leaf showing the placement of bulliform cells between a major and minor vein (bar = 20 μm). (g‐h) Transverse sections of (g) OE1 and (h) OE2 leaves showing a reduced size of bulliform cells (bar = 20 μm). (i‐j) Quantitative analysis of leaf traits in the Wt, OE1 and OE2 lines (i, j, k: *n* = 10). Bars represent the SD of measurements. Student’s *t*‐test was performed to determine significance: ** represents *P* < 0.01.

3′RACE (Rodriguez‐Cazorla *et al*., 2015) was conducted to define the 3′ end of the transcriptional unit of *Roc8* in wild type. The results indicated that the *crm1‐D* mutant carried a 50‐bp deletion immediately upstream of the poly(A) signal in the 3′UTR of *Roc8* (Figure 2c). The dominant nature of the *crm1‐D* mutant phenotype suggested that the sequences within the 50‐bp deletion might negatively regulate Roc8 protein expression in the wild type and might thus result in increased or ectopic expression of the Roc8 protein when deleted. To test this hypothesis, a polyclonal antibody was generated to the Roc8 peptide and used in Western blot analysis (Method S5). As shown (Figure 2d, f), Roc8 protein accumulated to approximately sevenfold higher levels in mutant leaf tissue relative to the wild type. To confirm that the overexpression of *Roc8* was responsible for the mutant phenotype, we introduced a 4.8‐kb BAC genomic fragment containing the full‐length coding and regulatory sequences of the wild‐type *CRM1* locus into *Nipponbare* callus (Figure 4a). A total of 50 independent transgenic lines were generated, and all phenocopied the original *crm1‐D* mutant in terms of plant stature (Figure 4b), leaf shape (Figure 4c, d, e), bulliform cell size (Figure 4f, g, h, j, k) and leaf rolling index (Figure 4i). The results strongly suggested that misexpression of *Roc8* resulted in the *crm1‐D* phenotype. To facilitate the description, we used *Roc8* to replace *CRM1* in subsequent studies.

### Roc8 is a transcriptional activator

As Roc8 belongs to an HD‐ZIP IV family member, it is predicted to encode a transcriptional activator but does not contain a classical nuclear localization signal. To test for subcellular localization of Roc8, green fluorescent signals were visualized using confocal microscopy (Chen *et al*., 2006). As expected, the GFP signal colocalized with DAPI fluorescence, indicating that Roc8 was exclusively targeted to the rice nucleus (Figure 3e, f, g, h, Method S7).

To decide whether Roc8 has transcriptional activation activity, the *Roc8* full‐length coding sequence was fused in frame with the pGBKT7 vector, and pGBKT7 was regarded as a negative control. Yeast cells containing pGBKT7‐Roc8 and the control were added to selective medium lacking Trp, His and Leu. As shown in Figure S5b, the clones (negative control) could not grow at all on medium lacking Trp, His and Leu. In contrast, the pGBKT7‐Roc8 strain exhibited gradual growth at 10^‐1^, 10^−2^ and even 10^−3^ dilutions and showed blue colour plus X‐α‐gal chromogenic reagent (Figure S5b). These results indicate that Roc8 has transcriptional activation activity.

Roc8 had three main motifs, containing the N‐terminus HD‐Zip domain (amino acids 1 to 200), START domain (amino acids 201 to 460) and C‐terminus (Figure S5a). To determine whether the N‐terminus, START domain or C‐terminus is responsible for transcription activation, Roc8 deletion vectors (Roc8‐A, B, C) fused with pGBKT7 were constructed. Among the three constructs, yeast cells containing the START domain only grew well (Figure S5b), indicating that the START domain of Roc8 may be responsible for its transcriptional activation activity.

### Roc8 3′‐UTR functions as a translational repressor

Three experiments were designed to verify *Roc8* 3′‐UTR function. First, GFP constructs containing different *Roc8* 3′‐UTRs were transiently transformed into rice protoplasts. GFP fluorescence, as well as GFP protein and mRNA levels, was tracked and compared to the control GFP‐Roc8 lacking the 3′‐UTR (Method S9). All the constructs localized to the nucleus, indicating that the Roc8 3′‐UTR did not influence Roc8 cellular localization. As expected, control GFP‐Roc8 was strongly expressed in the nucleus, whereas the constructs (GFP‐Roc8‐3′UTR‐FL and GFP‐Roc8‐50b) could significantly inhibit GFP fluorescence signals. Construct (GFP‐Roc8‐3′UTR‐△M) did not repress the GFP fluorescence signals in protoplasts under the same settings (Figure S6a). The reduction in GFP fluorescence was correlated with a reduction in GFP protein but not in GFP mRNA levels, as shown by Western blot and RT‐PCR analyses, respectively. The RT‐PCR experiment showed that the reduced GFP proteins were not caused by GFP mRNA levels (Figure S6b, c). The results indicate that *Roc8* 3′‐UTR 50‐bp sequence may have a post‐transcriptional regulation function.

Second, to explore whether the 3′‐UTR mediated Roc8 translation directly, we used the dual‐luciferase reporter assay in rice protoplasts to judge the Roc8 3′‐UTR effect (Method S10). The luciferase gene fused vectors bearing the Roc8 3′‐UTR deletion versions or the 35S terminator (35S Ter) were selected as reporters. A Renilla luciferase gene with a 35S terminator in its 3′‐UTR was cotransfected to normalize the transfection efficiency (Figure S7a). The ratio of relative luminescence/mRNA level was considered to estimate the relative translational efficiency. The results showed that the complete Roc8 3′‐UTR and the partial 3′‐UTR containing the 50‐bp sequence remarkably repressed luciferase activity, but the Roc8 3′‐UTR lacking the 50‐bp sequence had no obvious repression effect (Figure S7b). Furthermore, we replaced the 3′‐UTR 50‐bp sequence with an unrelated sequence (3′‐UTR‐50‐R1 or 3′‐UTR‐50‐R2). Compared to the complete 3′‐UTR (3′‐UTR‐FL), the ratio of the relative luminescence/mRNA level in the 3′‐UTR‐50‐R1 or 3′‐UTR‐50‐R2 constructs increased approximately threefold (Figure S7a, b). These results indicate that the Roc8 3′‐UTR 50‐bp sequence is a functional translational repression factor.

**Figure 5:**
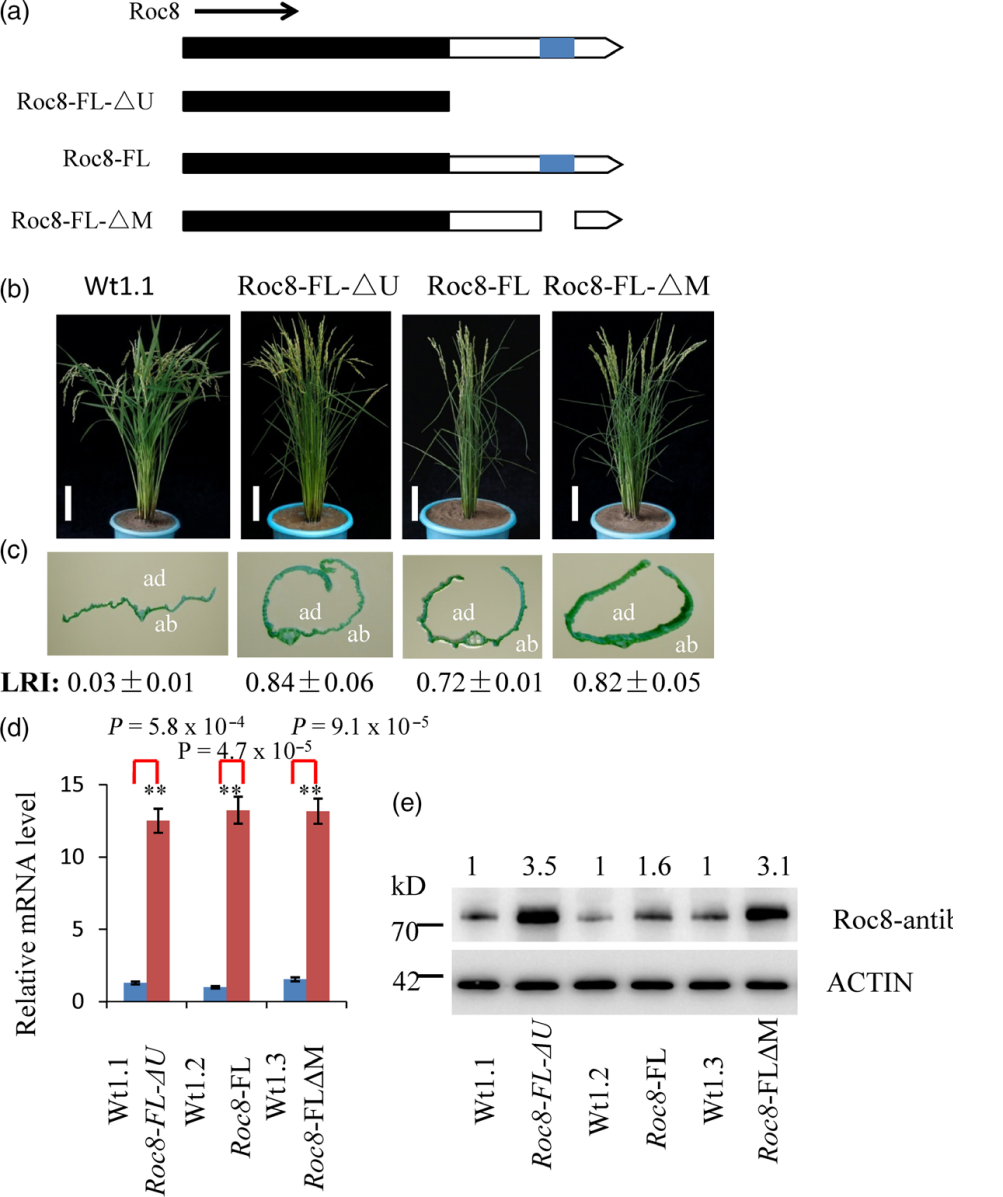
Features in the Roc8 3′‐UTR function as translational repressors. (a) Roc8 expression constructs. Transgenic expression of full‐length and deletion derivatives of the Roc8 gene are shown; the predicted CDS is in black, 3′‐UTRs are in white, and the 50 bp deletion found in the *crm1‐D* allele is shown in blue. All constructs were driven by the OsActin promoter as detailed in the Method S15. (b, c) Transgenic plants showing (b) representative T_1_ plants at maturity and (c) close‐up images of leaves and leaf rolling index of plant expression Roc8 vectors detailed above images (ab: abaxial side; ad: adaxial side). The nontransformed wild‐type (Wt1.1) control is shown at an equivalent developmental stage (bar = 5 cm). LRI: leaf erect index, *n* = 10. (d) Quantitative PCR assays of Roc8 mRNA levels in T_1_ transgenic and corresponding near‐isogenic null segregants (*n* = 3). Bars show the mean expression ± SD (**P < 0.01, Student's *t*‐test). (e) Semiquantitative analysis of Roc8 protein levels as measured by Western blotting using the Roc8 antibody in T_1_ transgenic lines and corresponding near‐isogenic null segregants. Actin was used as a loading control, and band intensities were quantified using ImageJ analysis of Western blots. The fold change shown above the blot is relative to Wt controls normalized by actin in each lane.

Third, a stable transformation experiment was used to test the *Roc8* 3′‐UTR functions (Method S15). Transgenic studies suggested that the 50‐bp deletion of the Roc8 3′UTR in *crm1‐D* resulted in the overproduction of *Roc8‐*encoded protein. The 50‐bp sequence comparisons suggested that 20 of 50 base pairs were conserved across multiple monocot and dicot species (Figure S8a, b). Furthermore, the deletion spanned a region required for a predicted stem–loop structure (Figure S8c). We speculated that a secondary stem–loop structure present in the 3′‐UTR of *Roc8* was required for efficient target repression and that deletion of this sequence results in ectopic expression of the *Roc8* transcript. To explore this hypothesis, we overexpressed the full‐length wild‐type *Roc8* gene with its native 3′‐UTR (Roc8‐FL), the full‐length *Roc8* gene including its 3′‐UTR (deletion 50 bp) (Roc8‐FL‐△M) and the complete coding sequence without the 3′‐UTR (Roc8‐FL‐△U) in the *Nipponbare* background (Figure 5a). As expected, the Roc8‐FL overexpression lines showed a semi‐rolled leaf phenotype (LRI: 0.72 ± 0.01; Figure 5b, c). However, the same transcript carrying a 50‐bp deletion in the 3′UTR (Roc8‐FL‐△M) resulted in a much stronger rolling phenotype (LRI: 0.82 ± 0.05), which was similar to plants carrying a construct that completely lacked the 3′ UTR region (Roc8‐FL‐△U) (LRI: 0.84 ± 0.06) (Figure 5b, c). Interestingly, equivalent levels of *Roc8* mRNA were observed in the Roc8‐FL and Roc8‐FL‐△M, Roc8‐FL‐△U overexpression lines (Figure 5d), but the Roc8 protein level was higher in the Roc8‐FL‐△M, Roc8‐FL‐△U (threefold increase compared to the wild type; Figure 5e). The above findings strongly suggest that the 50‐bp region in the *Roc8* 3′ UTR negatively regulates the expression of Roc8 activity and that the deletion of this sequence mediates the enhanced accumulation of Roc8 protein.

### Roc8 knockdown lines increased the size of bulliform cells and caused rolling outward

**Figure 6:**
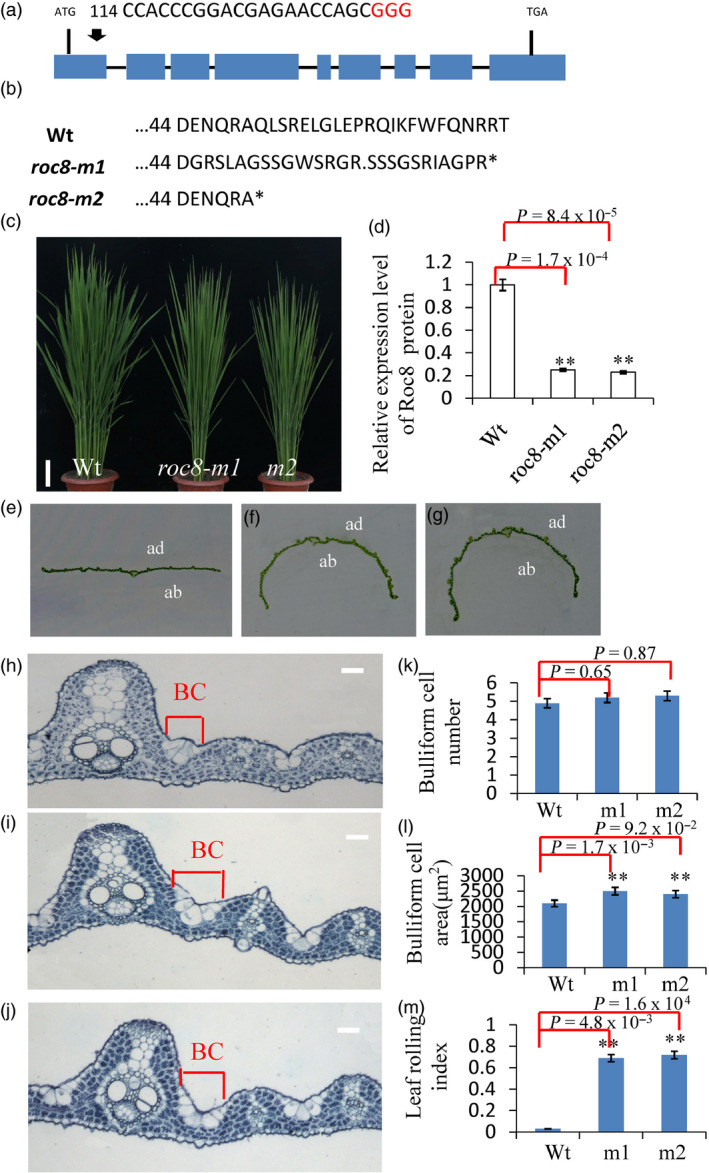
CRISPR/Cas9‐induced mutations in Roc8. (a) Schematic map of the genomic region of Roc8 and the sgRNA target site; arrow shows the sgRNA target site on the Roc8 genomic sequence; the PAM motif (NGG) is shown in red. Blue boxes represent Roc8 exons, and the black lines indicate intron sequences. (b) Amino acid alignment surrounding the sgRNA target region showing the predicted peptide sequence of wild‐type and mutant alleles. The site of the predicted frame‐shifted sequence is underlined, and new stop codons are shown by asterisks. (c) The phenotype of Wt, *roc8‐m1* and *roc8‐m2* homozygous mutants at the tillering stage (bar = 5 cm). (d) Quantitative PCR analysis showing Roc8 expression levels in Wt, *roc8‐m1* and *roc8‐m2* homozygous mutant plants. Actin was selected as an internal reference. (e‐g) Leaf phenotypes of (e) Wt, (f) roc8‐m1 and (g) roc8‐m2 homozygous mutants at maturity (ab: abaxial side; ad: adaxial side). (h‐j) Mature leaf showing the extent of bulliform cell area between a major and minor vein in (h) Wt, *roc8‐m1* (i) and *roc8‐m2* (j) homozygous mutants (bar = 20 μm). (k‐m) Bulliform cell characteristics in Wt and mutant plants, including (k) cell number, (l) area and (m) leaf rolling index (*n* = 10). Bars represent the SD of measurements. Student’s *t*‐test was performed to determine significance: ** represents *P* < 0.01.

Since *crm1‐D* is a gain‐of‐function mutant, a *Roc8* deletion mutation was created using CRISPR/Cas9 technology for further analysis. Two lines, *roc8‐m1* and *roc8‐m2*, contained a 10‐bp deletion in the 2nd exon (from 123‐132 bp) and a 5‐bp deletion in the 2nd exon of *Roc8* (from 138‐142 bp), respectively, causing early translation termination (Figure 6a, b). Quantitative PCR analysis in *roc8‐m1* and *roc8‐m2* plants determined that the Roc8 mRNA expression level was reduced significantly in the *roc8‐m1* and *roc8‐m2* plants, which indicated that *roc8‐m1* and *roc8‐m2* plants were knockdown mutants (Figure 6c, d). Furthermore, *roc8‐m1* and *roc8‐m2* plants showed an obvious rolling outward phenotype (Figure 6e, f, g), accompanied by increased bulliform cell size and leaf rolling index (Figure 6h, i, j, l, m) and normal bulliform cell numbers. The physiological measurements showed that *roc8‐m1* and *roc8‐m2* had higher photosynthesis, lower stomatal conductance, lower transpiration rate and lower Ci than the wild type (Figure S9a, b, c, d; Method S17). These results further suggest that Roc8 negatively regulates the size of bulliform cells.

### Double mutant analysis between Roc8 knockdown and Roc5 knockout mutants

Rice outermost cell‐specific genes (Roc) are transcription factors that play an important role in epidermal cell fate. *Roc5* negatively regulated bulliform cell fate and leaf development (Zou *et al*., 2011). Ito reported that the Roc family could form heterodimers to act (Ito *et al*., 2002). The knockdown mutants of *Roc5* and *Roc8* had similar leaf rolling towards the abaxial surface. We investigated whether Roc5 and Roc8 proteins interact *in vitro*. A yeast two‐hybrid assay verified that Roc5–AD could interact with Roc8–BD (Figure S9, Method S8). The double mutant was created by crossing *roc8‐m1* and *roc5* knockout mutant (Zou *et al*., 2011). F_1_ progeny showed a normal leaf shape phenotype. The double mutant was obtained by PCR amplification and DNA sequencing in F_2_ progeny. The leaf rolling degree in the double mutant was more serious than that in the single mutant (Figure S10a, b, c, d, e). The anatomical analyses in the double mutant (*roc5/roc8‐m1*) revealed that the number and size of the bulliform cells increased considerably compared to a single mutant (Figure S10f‐l). The expression analysis of the *Roc5* gene in the *crm1*‐*D* mutant or the expression analysis of the *Roc8* gene in *roc5* mutants showed no significant difference compared with wild type (Figures S14, S15). The above results indicated that *Roc8* and *Roc5* may function redundantly for the leaf rolling phenotype.

### Identification of Roc8 downstream pathways

Transcriptome analysis was used to determine *Roc8* downstream gene expression. Because *Roc8* expression was the highest at the 3rd leaf stage, triplicate mRNA samples of the *crm1‐D* plants and wild‐type plants were harvested (Method S6). Transcriptome data analysis indicated that 317 genes were up‐regulated and 251 genes were down‐regulated in *crm1‐D* plants (differentially expressed genes with more than twofold change (*P* < 0.01); Dataset S1). The differentially expressed genes mainly involved photosynthesis, organ compound metabolic process, generation of precursor metabolites and energy. Careful transcriptome data analysis identified that some genes related to the development of bulliform cells were inhibited in the *crm1*–D mutant (Table S2). Therefore, we selected five vacuolar cell developmental marker genes for qPCR experiments. The vacuolar cell development genes were generally inhibited (Figure S11). Additionally, some genes controlling bulliform cell development, such as *Roc5, NRL1, RL14, SRL1, ZHD1, OsMYB103L, AGO7* and *OsLBD3‐7,* were selected for qRT‐PCR detection in the *crm1‐D* mutant. The results showed that except for the *SRL1, OsMYB103L* and *OsLBD3‐7* genes, the expression levels of other genes were not significantly different in the *crm1‐D* mutant compared to the wild type (Figure S12). The above experiments indicated that *Roc8* may be involved independent of or partially dependent on the changed genes to regulate the development of bulliform cells.

The L1 box (AACATTTA) is well conserved within promoter regions of the regulated target genes of all L1‐specific HD‐Zip IV genes analysed to date (Ohashi., 2004; Zhang *et al*., 2010; Zou *et al*., 2011). Of all bulliform development‐related genes, only the promoter of the *OsLBD3‐7* gene (LOC_Os03g57670) contains an L1 box located approximately –487 to –494 bp from the transcription start site. The *OsLBD3‐7* expression level in *crm1‐*D increased more than threefold compared to the wild type (Figure S12).

**Figure 7:**
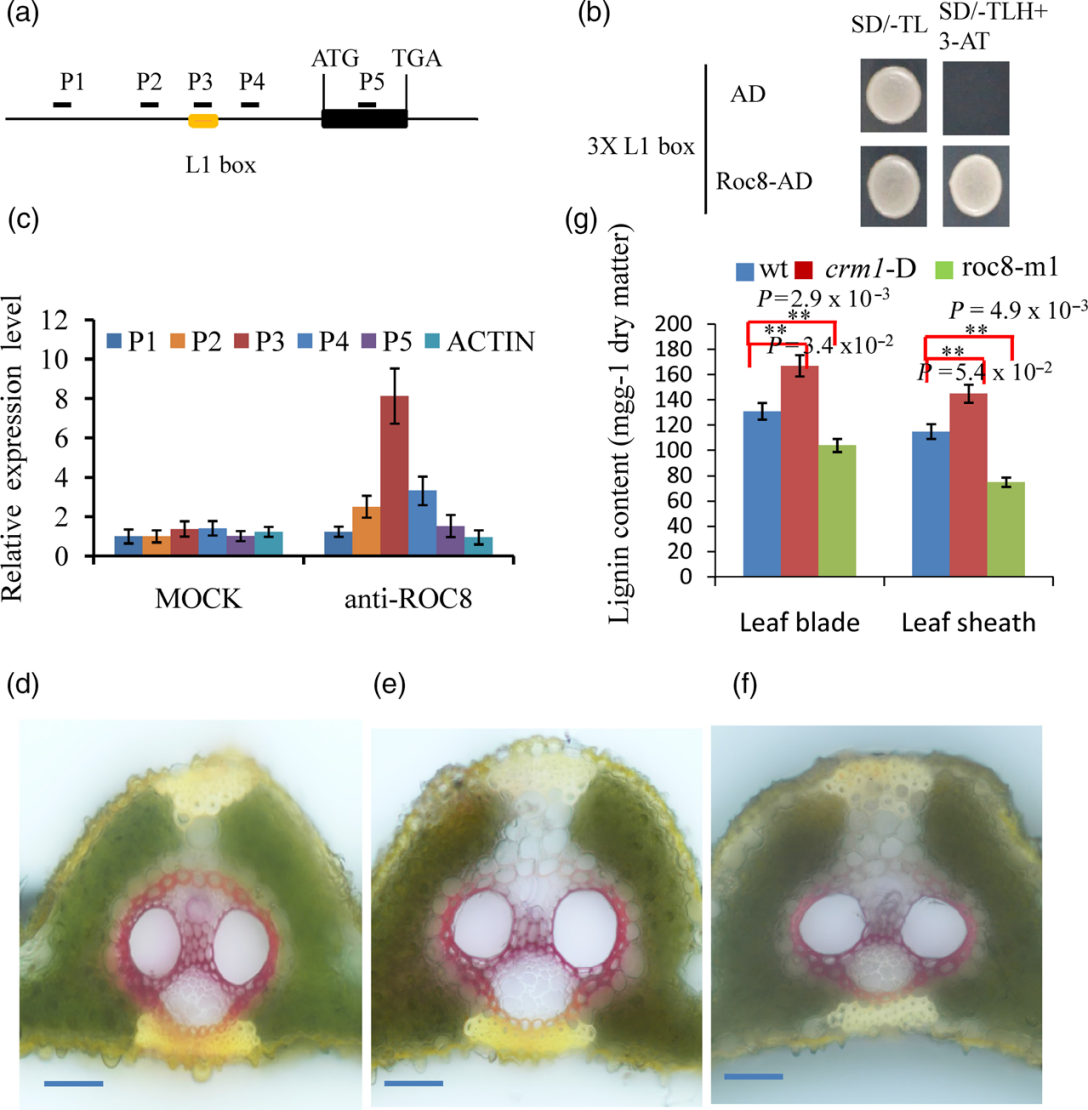
Roc8 regulated lignin content in rice leaves. (a) Schematic of the OsLAC17 promoter region showing regions of the promoter used for PCR amplification. The L1 box is located approximately −679— −617 base pairs upstream of the OsLAC17 start codon. (b) A yeast one‐hybrid assay was used to test Roc8 binding to the L1 box. The AD‐only vector was used as a control. (c) Anti‐Roc8 ChIP assays were performed on etiolated seedlings at the 3rd leaf stage. (d‐f) Histochemical analysis of lignin in cross sections of leaf vascular bundle using phloroglucinol staining in the (d) homozygous *crm1*‐D (e) Wt and (f) roc8‐m1 mutant. Bar: 25 μm. (g) Lignin contents were measured in mature leaf blades and sheath tissues (*n* = 3).

The *OsLBD3‐7* gene was reported to regulate the size of bulliform cells. Overexpression of *OsLBD3‐7* could induce adaxially rolled leaves in rice (Li *et al*., 2016). We sought to determine whether the *Roc8* gene could bind to the promoter of the *OsLBD3‐7* gene. First, vectors containing 3XL1 boxes and Roc8‐AD were constructed and imported into yeast. The strains could grow in SD medium (‐TLH + 3‐AT), but the control did not survive in SD medium (‐TLH + 3‐AT; Figure 7. b). Second, a luciferase activity assay was used to detect whether Roc8 can activate OsLBD3‐7. In the rice protoplasts cotransfected with the effector and reporter vectors, the ratio between LUC (firefly luciferase) and Ren (Renilla luciferase) of the effector OsLBD3‐7pro‐LUC was threefold higher than that of the empty vector control (Figure S13, Method S13). Third, a chromatin immunoprecipitation (ChIP) assay was performed using the Roc8 antibody (Method S14). As shown in Figure S14, the P3 region of the *OsLBD3‐7* promoter was more enriched than other regions using Roc8 antibody precipitation (Figure S14). The results showed that the *OsLBD3‐7* gene was one of the candidate targets of *Roc8* and *Roc8* as a transcriptional activator that positively regulated the size of bulliform cells by directly activating the *OsLBD3‐7* gene.

### *Roc8* knockdown mutant reduced lignin contents without production penalty

Notably, many genes involved in cell wall lignin formation metabolism and cell wall cellulose formation also displayed a marked change in *crm1‐D* mutants in differentially expressed data (Table S2). Several celluloses and lignin‐related genes were selected for qPCR tests. The results further verified that cellulose formation and lignin synthesis genes were greatly increased in the *crm1‐D* mutant (Figure S16a, b). The promoters of these genes were used to determine whether they contain the L1 box. Among these genes, only the *OsLAC17* and *OsLAC22* promoters contained the L1 box, ranging from −679 to −671 bp and from −1178 to −1170 bp from the transcription start site, respectively. We speculated that the *OsLAC17* and *OsLAC22* genes may be potential target genes of Roc8. Li reported that *OsLAC17* was responsible for lignin biosynthesis (Li *et al*., 2017); thus, *OsLAC17* was selected as a candidate gene for subsequent analysis. We further tested whether Roc8 could bind to *OsLAC17* chromatin to mediate lignin biosynthesis. First, a luciferase activity assay was used to detect whether Roc8 can activate *OsLAC17*. In the rice protoplasts cotransfected with the effector and reporter vectors, the ratio between LUC (firefly luciferase) and Ren (Renilla luciferase) of the effector *OsLAC17*pro‐LUC was significantly threefold higher than that of the empty vector control (Figure S13). Second, a chromatin immunoprecipitation (ChIP) qPCR assay was performed using the Roc8 antibody. As shown in Figure 7a, c, the P3 region of the *OsLAC17* promoter was more enriched than other regions using Roc8 antibody precipitation. The results showed that the *OsLAC17* gene was also one of the candidate targets of *Roc8* and was activated by Roc8.

To further confirm whether the lignin changed in the mutant, the phloroglucinol dye assay was used to display lignin changes. The phloroglucinol–HCl staining assay showed stronger red staining of the leaf vascular bundle in the *crm1‐D* mutant and weak red staining of the leaf vascular bundle in *roc8* knockdown lines compared to the wild type (Figure 7d, e, f). Next, leaf blade/sheath lignin contents in the *crm1‐D* mutant and the wild type were measured (Method S12). The *crm1‐D* mutant retains a significantly higher lignin content in leaves (Figure 7g). In *roc8* knockdown lines, the lignin contents of the leaf blade and sheath were reduced to 75%–85% of the wild type. Notably, *roc8* knockdown lines m1 and m2 showed excellent agronomic traits, such as normal height, normal seed set rate (Figure 6, Table S1), higher photosynthetic efficiency, lower transpiration rate and higher 1000‐grain weight (Figure S17, Table S1, Method S16). Taken together, the results of this study indicate that *Roc8* also positively mediated lignin biosynthesis without the production penalty. This result indicated that knocking down the *Roc8* homologous gene in forage grass may have strong practical application prospects in cultivating low lignin materials without a production penalty.

## Discussion

The epidermis is the outermost cell layer of a plant leaf and provides a barrier that allows maintenance of leaf water homeostasis (Martin and Glover, 2007). Epidermal cells are specially differentiated cells and have different cell types, such as bulliform cells, cuticular trichome structures and stomata. Extensive studies have established regulatory mechanisms controlling epidermal cell fate in *Arabidopsis* and other species (Cheng *et al*., 2014; Le *et al*., 2014; Takada and Iida, 2014; Takada *et al*., 2013; Wang and Chen, 2014). Bulliform cells belong to the monocotyledon‐specific epidermal cell structure. Only by studying monocotyledon model plant leaf morphology can we elucidate this kind of cell regulation mechanism. At present, much progress has been made in regulating the development of bulliform cells (Xu *et al*., 2018). However, the fine regulatory network governing this process has not been elucidated. In this study, a constitutively rolling leaf mutant, *crm1*‐D, was obtained from our previous rice mutant population (Wan *et al*., 2009; Zou *et al*., 2014), and the corresponding cloned gene was *Roc8* (Figure 2), a member of the HD‐Zip IV family. Sequence analysis revealed that the 50‐bp deletion occurred in the *Roc8* 3′‐UTR in *crm1*–D (Figure 2). A series of experiments showed that the 50‐bp sequence in the Roc8 3′‐UTR represses *Roc8* at the translational level *in vitro* and *in vivo* (Figures 5, S6, S7). The size of bulliform cells was increased in *Roc8* knockdown lines (Figure 6). Moreover, a series of assays, including the transcriptome profile, qPCR analysis, yeast two‐hybrid assays, luciferase activity detection and ChIP experiments, confirmed that *Roc8* acted as a transcriptional activator and bound to the *OsLBD3‐7* promoter, regulating the size of bulliform cells (Figures S12,S13,S14). Therefore, our work showed that Roc8 plays an essential role in the formation and development of epidermal bulliform cells in rice. HD‐Zip IV family genes seem to have a conserved function in regulating epidermal cell fate both in dicots and monocots.

### Roc8 and Roc5 play a redundant role in regulating the size of bulliform cells in rice

Leaf rolling is a simple and general phenotype that is easy to observe by the naked eye and thus has received more attention from traditional breeders. However, this phenotype is not due to a simple development process and is regulated by multiple tissue cells. At present, there are many factors that affect leaf rolling in rice, including changes in the number and size of bulliform cells, programmed cell death of sclerenchyma cells on the adaxial/abaxial surface (Zhang *et al*., 2009) and cuticle development (Wu *et al*., 2011). Bulliform cells generally occur intercostally as long strips several cells wide. Bulliform cells aligned at the abaxial side with linear patterning by positional information in maize, similar to the trichome pattern in *Arabidopsis* (Hernandez *et al*., 1999). In *Arabidopsis*, trichomes are single cells and never linked to each other; bulliform cells are arranged in clusters of several cells, and the clusters are not adjacent to each other in rice. Our study revealed that Roc8, a member of the rice outermost cell‐specific gene (Roc) family, plays an important role in epidermal cell fate. At present, Roc1, Roc4 and Roc5 have been clarified in detail. The expression of *Roc1* in the outermost cells may be dependent on the positional information of cells in the embryo or meristemoid cells prior to cell fate determination of the protoderm (epidermis; Ito *et al*., 2002). Roc4 negatively regulates cuticular wax biosynthesis by E3 ligase degradation in rice (Wang *et al*., 2018). In contrast to Roc1 and Roc4, Roc8 and Roc5 play a redundant role in regulating the size of bulliform cells in rice. First, overexpression in Roc8 and Roc5 caused leaf rolling with adaxially curved leaves. Suppression in Roc8 and Roc5 caused leaf rolling with abaxially curved leaves. Second, the size of bulliform cells increased in Roc8 knockdown and Roc5 knockout plants but was reduced in Roc5‐overexpressing lines and *crm1*‐D lines. Third, Roc5 and Roc8 have similar expression patterns. Roc5 is mainly expressed in the L1 layer of the meristem but not in mature leaves (Ito *et al*., 2002). *In situ* hybridization assays showed that Roc8 was expressed in epidermal cells and vascular bundles (Figure 3), suggesting that Roc5 or Roc8 may participate in the establishment of bulliform cells, rather than their maintenance in the adaxial epidermis. Fourth, the yeast two‐hybrid assays showed that Roc5 interacted with Roc8 *in vitro*, which was consistent with Roc family members acting as heterodimers (Ito *et al*., 2002). Fifth, cytology analysis showed that the size of bulliform cells in the double mutant was significantly increased compared with that of the single mutant.

Although the two genes Roc5 and Roc8 are functionally redundant in controlling the development of bulliform cells, the developmental processes controlling cell expansion and division may be slightly different. First, the Roc8 expression pattern was slightly different from the Roc5 expression pattern. From the Roc8 *in situ* hybridization pattern (Figure 3) and Roc5 GUS analysis (Zou *et al*., 2011), Roc8 expression began earlier than Roc5 expression. Roc8 expression began before the 3^rd^ stage, whereas Roc5 expression began at the 3rd stage. This finding is consistent with the period of the respective phenotype. Second, Roc8 mainly controls the size of bulliform cells, while Roc5 primarily controls the number and size of bulliform cells. We speculate that Roc8 may be the main regulator of bulliform cell expansion, while Roc5 may be the regulator of bulliform cell division. Quantitative PCR analysis showed that in the roc8‐m1 and roc8‐m2 lines (knockdown mutants), the expression of some genes controlling cell expansion, such as OsXTH9, OsXTH11, OsEXPA2, OsEXPA4, OsEXPA5 and OsEXPB11, increased significantly (Figure S17a), while in the roc5 mutant, some genes controlling the cell cycle, such as the cell cycle proteins OsCycA1;2 and OsCycA2;1, increased significantly (Figure S17c). Additionally, the expansion genes, such as OsEXPA2, OsEXPA4, OsEXPB11 and OsXTH11, increased slightly in roc5 (Figure S17a), which also indicated that Roc8 and Roc5 were partially redundant in controlling the size of bulliform cells.

### Potential regulation mechanism of bulliform cell expansion

Although we have found two direct target genes, how could Roc8 regulate the cell expansion of bulliform cells in rice? We think that Roc8 can regulate the expansion of bulliform cells by the following possible mechanisms. First, the expansion of bulliform cells needs to change the composition of the cell wall. The *nrl1* mutant showed reduced leaf width, half‐rolled leaves and dwarfing. The *nrl1* mutant had a smaller bulliform cell size. The *Nrl1* gene encodes the cellulose synthetase gene (Hu *et al*., 2010). Roc8 can induce the expression of xyloglucan endotransglucosylase/hydrolases (XTHs) and expansion genes, and whether Roc8 directly binds with its promoter needs to be further verified. Second, gibberellin biosynthesis and auxin biosynthesis are involved in the developmental process of bulliform cells. YAB1 was involved in feedback regulation of GA3 biosynthesis in rice. The YAB1 RNAi plants rolled abaxially to form a cylinder‐like structure (Dai *et al*., 2007). Nal7 encodes a riboflavin monooxygenase of the YUCCA family member. The *nal7* mutant showed narrowed leaves and reduced auxin contents. The number and size of bulliform cells in *nal7* were reduced, resulting in the leaf rolling phenotype (Fujino *et al*., 2008). The *cow1* mutant, an allele of Nal7, results in rupture of the largest bulliform cell and consequently rolled leaves (Woo *et al*., 2007). In the transcriptome data and quantitative PCR analysis, auxin synthesis‐related genes showed significant changes in the mutant. Roc8 may regulate the size of bulliform cells by affecting auxin synthesis genes (Figure S18b) but is not involved in the gibberellin biosynthesis pathway (Figure S19). Third, the acid growth theory was used to explain cell expansion (Moloney *et al*., 1981). Roc8 activated OsLBD3‐7. Lateral organ boundary domain families (LBDs) and auxin‐responsive genes (ARFs) are often involved in auxin signalling pathways (Kim and Lee, 2013). Deregulation of the OsmiR160 target gene OsARF18 leads to leaf rolling in rice by affecting auxin signalling (Huang *et al*., 2016). Auxin activated the PMAs and acidified the intercellular space. Changes in apoplastic pH activity result in higher turgor pressure between the outer and inner membranes, thereby enabling cell expansion (Hager., 2003). Roc8 may affect cell expansion in all the above ways, and the identification of direct Roc8 target genes should help to clarify this question.

### Roc8 3′‐UTR functions as a translational repressor

The 3′‐UTR plays an important role in the growth and development of animals and plants. At present, the mechanism of this region primarily affects transcription level, post‐transcriptional modification and protein translation (Li *et al*., 2015; Shen *et al*., 2017). Extensive evidence supports a 3′‐UTR as a regulatory factor that could influence the expression of adjacent genes (Kumar, 2014; Silveyra *et al*., 2014). The 3′‐UTR could alter adjacent gene transcription through epigenetic modifications (Mao *et al*., 2015; Wei *et al*., 2014). We first analysed the effect of *crm1‐D* on *Roc8* DNA methylation using bisulphite genomic sequencing. The CG, CHG and CHH methylation levels were unchanged (Figure S20 and Method S11). This finding supported the conclusion that the mRNA level of *Roc8* in *crm1‐D* did not vary and that the 3′‐UTR did not regulate *Roc8* gene expression at the transcriptional level. Shen reported a translational repression mechanism mediated by a stowaway‐like MITE (sMITE) site embedded in the 3′‐UTR of Ghd2 (Shen *et al*., 2017). Klein‐Cosson provided evidence for the existence of a 24‐nt small RNA, which was complementary to the 3′‐UTR of OCL1 (Outer Cell Layer1; Klein‐Cosson *et al*., 2015). These researchers argued that it was possible for a secondary stem–loop structure present in the 3′ UTR of *OCL1* to be an efficient target for repression. The 3′‐UTR of *OCL1* not only influences the *OCL1* mRNA level but also affects the translational level (Klein‐Cosson *et al*., 2015). However, this conclusion lacked more genetic data to support this hypothesis. In this study, using a series of *in vitro* and *in vivo* experiments, we verified that the Roc8 3′‐UTR does not only affect the expression of RNA but also affects the protein level. 3′‐UTR analyses of Roc8, Roc5, Roc4, Roc6, ZmOCL1, ZmOCL4, AtANL2 and AtHDG1 (Figure S8) showed that a conserved 20‐bp sequence exists in HD‐Zip IV families, indicating that some gene duplications in dicots and monocots occurred before their divergence.

We further analysed the possible regulation mechanism of the 50‐bp sequence as follows. (i) This region may be siRNA or miRNA target sites, similar to OCL1 in rice (Klein‐Cosson *et al*., 2015). We predicted that the 3′ UTR of *Roc8* contained a miRNA‐ or siRNA‐binding region, but there were no relevant miRNAs or siRNAs complementary to the 3′ UTR of *Roc8* in the current database. The search for miRNA or small RNA precursors targeting the 3′ UTR of *Roc8* would be a target for future research. (ii) This region may be a transposon regulatory region, and some of the smaller transposons are small reverse repeat transposon elements whose transcription instinct forms a stable stem–loop structure. (iii) The stem–loop structure may serve as a termination element, which may be similar to poly(A) as a termination signal (Wang *et al*., 2019). Roc8, as a transcription factor, may regulate multiple physiological processes of plant development. Finely controlling the Roc8 mRNA or protein level is necessary to improve plant adaptability. The next step is to determine which factors affect the binding of this target region and whether other RNA‐binding proteins (RBPs) or regulatory factors are needed to participate in this process of translation repression.

### Roc8 positively regulated leaf lignin biosynthesis

Lignin is a complex organic polymer that forms important structural materials in vascular plants and some algae supporting tissues (Vanholme *et al*., 2012). Lignin plays an important role in the structure and repair mechanism of the plant cell wall, and scientists hope to modulate lignin contents by genetic engineering (Halpin, 2019). In this study, by analysing the transcriptome data, some controlling lignin biosynthesis genes increased in *crm1‐D*. Five additional experiments confirmed the hypothesis that *Roc8* positively regulated leaf lignin contents. First, qPCR verified that the *OsLAC17* expression level was significantly increased in the *crm1‐D* mutant. Second, a chromatin immunoprecipitation (ChIP) assay using the Roc8 antibody in wild‐type protoplasts confirmed that *Roc8* could directly bind to the promoter L1 region of the *OsLAC17* gene. Third, in the rice protoplasts cotransfected with the effector and reporter vectors, the ratio between LUC (firefly luciferase) and Ren (Renilla luciferase) of the effector *OsLAC17*pro‐LUC was significantly (threefold) higher than that of the empty vector control. Fourth, the phloroglucinol histochemical analysis in leaf vascular bundle cross sections showed that there was a higher signal in the walls of the xylem cells in the *crm1‐D* mutant and a lower signal in the *Roc8* knockdown lines compared to wild‐type plants. Fifth, the lignin content measurements showed that there were higher lignin contents in *crm1‐D* and lower lignin contents in *Roc8* knockdown lines. The above five experiments strongly verified that *Roc8* positively regulated the lignin contents in rice.

Many transcription factors, such as *OsTF1L, OsIDD2* and *OsMYB103L*, participate in the regulation of lignin biosynthesis in rice. Overexpression of *OsTF1L*, a rice HD‐Zip transcription factor, promoted lignin biosynthesis (Bang *et al*., 2019). *OsIDD2*, a zinc finger and indeterminate domain protein, regulates secondary cell wall formation (Huang *et al*., 2018). *OsMYB103L*, an R2R3‐MYB transcription factor, influenced leaf rolling and mechanical strength in rice (Yang *et al*., 2014). Simultaneously, overexpression of these genes often caused unfavourable developmental defects, such as stunted growth, dwarf, brittle culms and decreased fertility, which limited their application prospects. Notably, the lignin contents decreased slightly in *Roc8* knockdown lines (75%–85%). These lines not only did not exhibit adverse agronomic traits but also showed high photosynthetic efficiency due to plant keeping moderate rolling, making the leaves upright, more leaves exposed to light and enhancing the light quantity and intensity at the base of the canopy, suggesting that the use of *Roc8* or homologous genes in the production of materials with lower lignin content has important practical application value.

The results of this study support a proposed model illustrating the *Roc8* mechanism (Figure S21). We verified the regulatory function of the 3′‐UTR of *Roc8* by forward genetics and provided substantial evidence to clarify Roc8 function by regulating the size of bulliform cells by activating the *OsLBD3‐7* gene. In addition, *Roc8* bound to the promoter of the *OsLAC17* gene, which could be involved in modulating lignin biosynthesis. By reverse genetics, we created *Roc8* knockdown lines using gene editing technology and revealed that the *Roc8* knockdown lines not only improved the photosynthetic efficiency because of leaf morphology change from the large size of bulliform cells but also had lower lignin contents without the production penalty. Future research may obtain functional clues for combining other Roc homologous genes to cultivate high photosynthetic efficiency crops in rice or low lignin contents in forage grass.

## Materials and methods

### Material screening and growth condition

In previous research, a rice mutant population was constructed using the *Japonica* cultivar *Nipponbare* as the recipient (Wan *et al*., 2009). The *crm1‐D* mutant was screened from this population. Genetic analysis was conducted by cross test between *crm1‐D* and *Nipponbare* (wild type). A map of the F_2_ population was created from a cross between *crm1‐D* and *Dular* (*Indica*). The materials were grown under natural growth conditions in a paddy field (Zou *et al*., 2014).

### Positional cloning of *Roc8*

An initial set of *crm1‐D*/Dular F_2_ progeny was used for coarse linkage analysis; fine mapping was then performed based on 890 homozygous mutant F_2_ segregants. The polymorphic InDel markers used to genotype these progenies are presented in Table S3. The positional cloning strategy was previously described (Zhang *et al*., 1994). The amplicons were separated on 6% polyacrylamide gels and visualized by silver staining. Finally, products in the candidate region were amplified, sequenced and aligned. The mutant region was verified using primer pairs p1/p2 between wild‐type and *crm1‐D* DNA or cDNA.

### Recapturing the *crm1‐*D phenotype

BAC clone P0664C05 containing the *Roc8* gene was first cut by *Pst*I and *EcoR*I restriction enzymes. The product carrying a 1.8 kb *Roc8* native promoter, full‐length *Roc8* coding region and 1 kb 3′‐UTR were ligated into *p*Cambia2300 with the selectable marker *NPT*II. The expression vector was introduced into the *Agrobacterium* tumefaciens strain AGL1 using the heat‐shock method and then transformed into *Nipponbare* calli. The regenerated plant leaves in T_1_ progeny were harvested, and histological observations were conducted. The areas and numbers of bulliform cells were numbered as mentioned above. Ten independent visual fields were counted (*n* = 10).

### Creation of the *Roc8* knockdown lines

*Roc8* knockdown plants were generated using CRISPR/Cas9. The sgRNAs were designed to target *Roc8* locations. The single sgRNA was created in the BGK03 vector containing Cas9, which was introduced into *Agrobacterium* strain EHA105 and transformed into *Nipponbare*. Eight independent lines of sgRNA were obtained. To examine the function of CRISPR/Cas9 in vivo, genomic DNA was extracted from transgenic plants, and primer pairs flanking the designed target site were used for PCR amplification (Table S3). Sequence alignment revealed that *Roc8* knockdown lines were obtained.

### Phloroglucinol–HCl staining test

Fresh mature leaves were collected and carefully cut with razor blades. The leaves were moved to the slide and stained with phloroglucinol (1%) staining liquid for two minutes. After staining, concentrated hydrochloric acid was added to the slide (Srivastava, 1966). The photographs were taken with a light microscope (Zeiss). (*n* = 3).

### Histology and cytology observation

Histology, cytology, TEM analysis and *in situ* hybridization were described in Methods S1, S2 and S3.

### Statistical analysis

Bars show the mean value ± s.d. Student’s *t*‐test analysis was used to test for significant differences between the above lines (*P < 0.05, **P < 0.01).

### Primer sequences

The primers used in this study are listed in Table S3.

## Conflicts of interest

The authors declare no conflicts of interest.

## Author Contribution

Z.G.Z. and L.T.G designed the experiments and wrote the manuscript. S.J, C.X.A and T.S.Z performed map‐based cloning of *Roc8*. Z.K.L performed the gene expression analysis. W.Y.W performed ChIP experiment and Western blotting test. C.Z.H performed subcellular localization assays. W.J.X, S.X.H and A.P.F conducted the transgenic experiment. W.P.Q gave critical advice. Z.G.Z and T.G.L designed the total experiments.

## Supporting information


**Figure S1** Photosynthesis parameter measurements from the *crm1*‐D plant and wild type at the flowering stage.
**Figure S2** Toluidine blue O staining and scanning electron microscopy observations of cross‐sections of seedling leaves in *Oryza sativa*.
**Figure S3** Comparison of transcript abundance of candidate genes in wild‐type (Wt) and *crm1*‐D mutant by qRT–PCR.
**Figure S4** Expression pattern analysis of Roc8 at the seedling stage and mature stage.
**Figure S5** Protein structure, transactivation analysis of Roc8.
**Figure S6** Fluorescence detection of GFP‐Roc8 3′‐UTR with different fusion constructs using a rice protoplast transient transformation system.
**Figure S7** Roc8 3′‐UTR regulates luciferase activity.
**Figure S8** Roc8 3‐UTR sequence analysis.
**Figure S9** A yeast two‐hybrid interaction assay was performed with Roc5 and Roc8.
**Figure S10** Double mutant analysis of loss‐of‐function alleles of roc5/roc8.
**Figure S11** Expression analysis of vacuolar development‐related genes.
**Figure S12** Bulliform cell development‐related gene expression analysis in rice.
**Figure S13** LUC transient transactivation assay in rice protoplasts.
**Figure S14** Chip experiment for Roc8 binding to the promoter of the OsLBD3‐7 gene.
**Figure S15** Expression analysis of Roc8 in the Roc5 mutant and wild type at the 3rd leaf stage.
**Figure S16** Expression analysis of cellulose‐related genes and lignin biosynthesis genes in rice.
**Figure S17** Photosynthesis parameter measurements from the roc8‐m1 plant and wild‐type plant at the late tillering stage.
**Figure S18** RT‐qPCR expression analysis of cell expansion and cell division genes.
**Figure S19** RT‐qPCR expression analysis of the gibberellin biosynthesis pathway genes.
**Figure S20** Distribution of three cytosine contexts and methylation patterns in the ∼800‐bp promoter region of Roc8 between wild type and crm1‐D.
**Figure S21** Schematic of the hypothesis illustrating the possible mechanism underlying Roc8 in this study.
**Table S1** Agronomic trait comparison between wild type, crm1‐D and Roc8‐m1 mutants at the mature stage.
**Table S2** Differentially expressed proteins associated with cellulose, lignin, epidermis development in leaves.
**Table S3** Primers used for fine mapping, qRT‐PCR, vector construction and etc.
**Methods S1** Transmission Electron Microscopy (TEM) Analysis.
**Methods S2** Histology and cytology observation.
**Methods S3** In situ hybridization.
**Methods S4** RNA preparation and quantitative real‐time (qRT)‐PCR analysis.
**Methods S5** Protein extraction, sodium dodecyl sulfate polyacrylamide gel electrophoresis (SDS‐PAGE), and Western blotting.
**Methods S6** Transcriptome data analysis.
**Methods S7** Subcellular localization of Roc8 in rice.
**Methods S8** Yeast two‐Hybrid assay and Yeast one‐hybrid assay.
**Methods S9** Rice protoplast transformation assay.
**Methods S10** Luciferase assay to detect Roc8 3′‐UTR function using a rice protoplast transient transformation system.
**Methods S11** Bisulfite sequencing analysis.
**Methods S12** Lignin content measurement.
**Methods S13** Luciferase transient transcriptional activity assay in rice protoplasts.
**Methods S14** Chromatin immunoprecipitation (ChIP) and quantitative PCR analysis.
**Methods S15** Roc8 3′‐UTR deletion construction.
**Methods S16** Field evaluation for agronomic traits.
**Methods S17** Photosynthetic parameters measurement.

**Dataset S1** Differentially expressed genes in crm1‐D and wild type.
