# Supplementary material for: HD‐ZIP IV gene Roc8 regulates the size of bulliform cells and lignin content in rice
Source: Plant Biotechnol J. 2020 Jul 8;18(12):2559–72. doi: 10.1111/pbi.13435 (PMC7680540; doi:10.1111/pbi.13435)
Supplement: Supplementary file 1 — Figure S1 Photosynthesis parameter measurements from the crm1‐D plant and wild type at the flowering stage. Figure S2 Toluidine blue O staining and scanning electron microscopy observations of cross‐sections of seedling leaves in Oryza sativa. Figure S3 Comparison of transcript abundance of candidate genes in wild‐type (Wt) and crm1‐D mutant by qRT–PCR. Figure S4 Expression pattern analysis of Roc8 at the seedling stage and mature stage. Figure S5 Protein structure, transactivation analysis of Roc8. Figure S6 Fluorescence detection of GFP‐Roc8 3′‐UTR with different fusion constructs using a rice protoplast transient transformation system. Figure S7 Roc8 3′‐UTR regulates luciferase activity. Figure S8 Roc8 3‐UTR sequence analysis. Figure S9 A yeast two‐hybrid interaction assay was performed with Roc5 and Roc8. Figure S10 Double mutant analysis of loss‐of‐function alleles of roc5/roc8. Figure S11 Expression analysis of vacuolar development‐related genes. Figure S12 Bulliform cell development‐related gene expression analysis in rice. Figure S13 LUC transient transactivation assay in rice protoplasts. Figure S14 Chip experiment for Roc8 binding to the promoter of the OsLBD3‐7 gene. Figure S15 Expression analysis of Roc8 in the Roc5 mutant and wild type at the 3rd leaf stage. Figure S16 Expression analysis of cellulose‐related genes and lignin biosynthesis genes in rice. Figure S17 Photosynthesis parameter measurements from the roc8‐m1 plant and wild‐type plant at the late tillering stage. Figure S18 RT‐qPCR expression analysis of cell expansion and cell division genes. Figure S19 RT‐qPCR expression analysis of the gibberellin biosynthesis pathway genes. Figure S20 Distribution of three cytosine contexts and methylation patterns in the ∼800‐bp promoter region of Roc8 between wild type and crm1‐D. Figure S21 Schematic of the hypothesis illustrating the possible mechanism underlying Roc8 in this study. Table S1 Agronomic trait comparison between wild type, crm1‐D and Roc8‐ [file PBI-18-2559-s002.pdf]

SUPPLEMENTAL FIGURES:

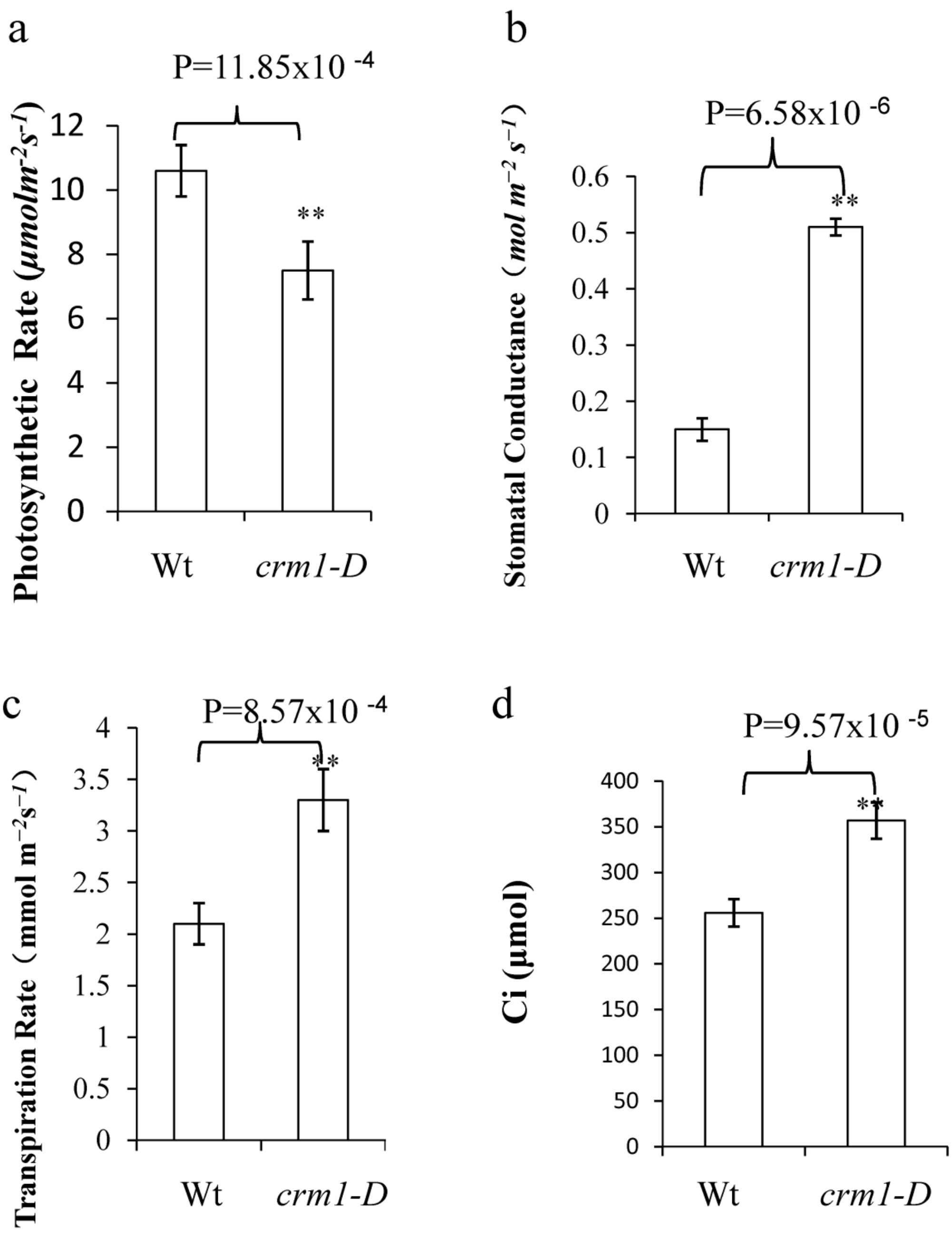

Figure S1. Photosynthesis parameter measurements from the *crm1*-D plant and wild type at the flowering stage.

a: Photosynthetic rate comparison. **b:** Stomatal conductance comparison. **c:** Transpiration rate comparison. **d:** Ci comparison. Bars represent the SD of three measurements. Student’s t-test was performed using the raw data; two asterisks indicate statistically significant differences at $P<0.01$.

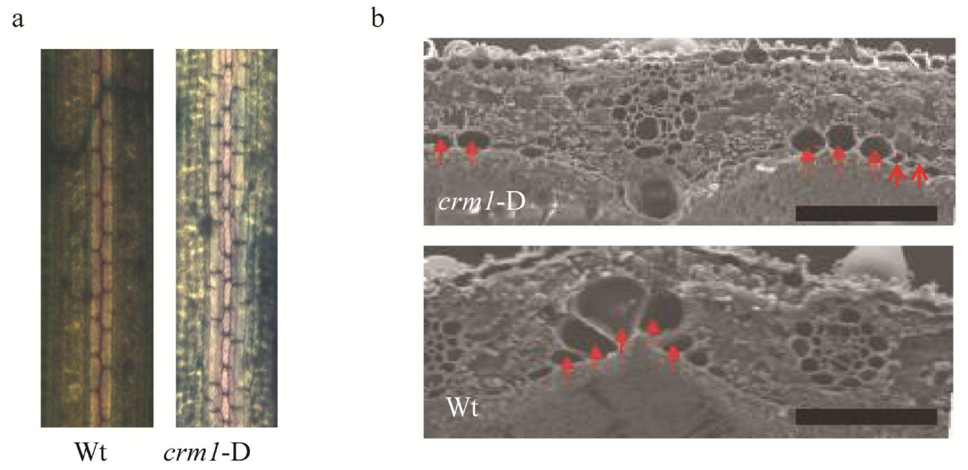

Figure S2. Toluidine blue O staining and scanning electron microscopy observations of cross-sections of seedling leaves in *Oryza sativa*.

a: Adaxial epidermal peels abutting the small veins of wild type (Wt) and *crm1*-D using toluidine blue O staining.

b: Scanning electron microscopy for cross section of seedling leaves of wild type (Wt) and *crm1*-D. Red arrows denote the bulliform cells. Bar=100 μm.

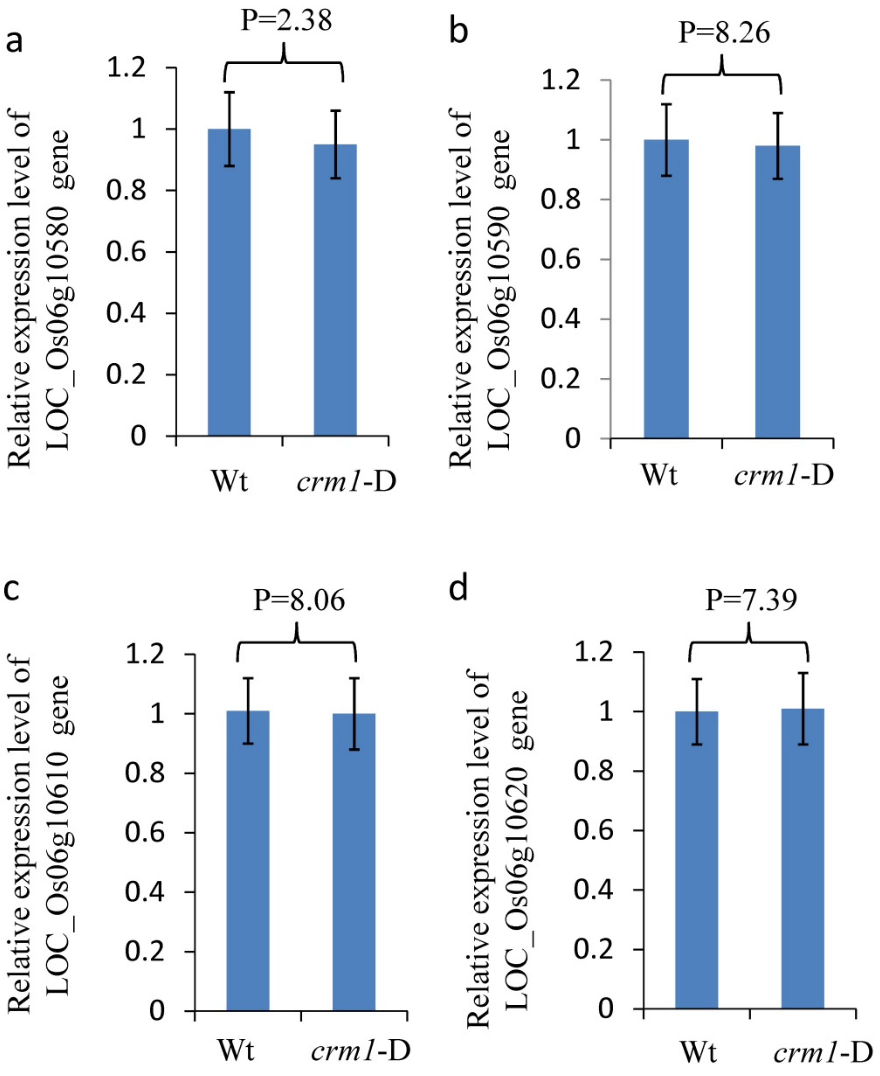

Figure S3. Comparison of transcript abundance of candidate genes in wild-type (Wt) and *crm1*-D mutant by qRT–PCR.

a. Relative expression level of LOC_Os06g10580 gene analysis between wild-type (Wt) and *crm1*-D plants.

b. Relative expression level of LOC_Os06g10590 gene analysis between wild-type (Wt) and *crm1*-D plants.

c. Relative expression level of LOC_Os06g10610 gene analysis between wild-type (Wt) and *crm1*-D plants.

d. Relative expression level of LOC_Os06g10620 gene analysis between wild-type (Wt) and *crm1*-D plants. Bars represent the SD of three measurements.

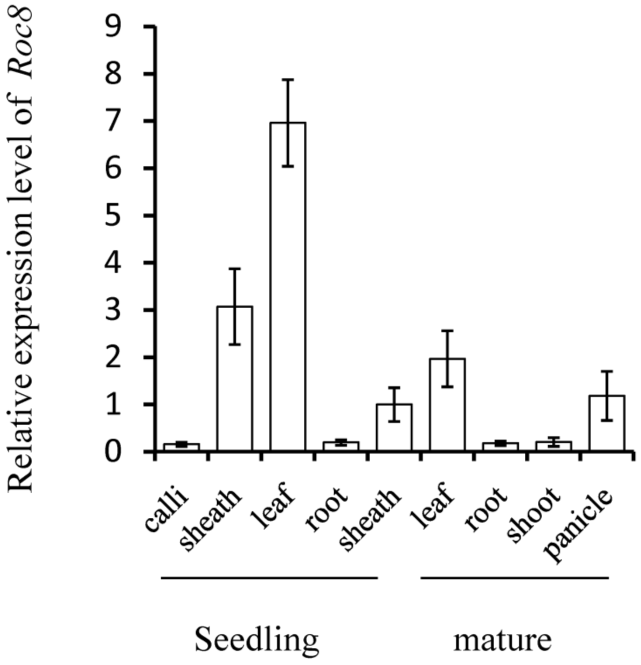

Figure S4. Expression pattern analysis of Roc8 at the seedling stage and mature stage. An expression analysis was conducted by qRT-PCR using RNA samples of wild-type plants prepared from the seedling and mature stages. The results showed that Roc8 preferred the highest expression in the young leaves.

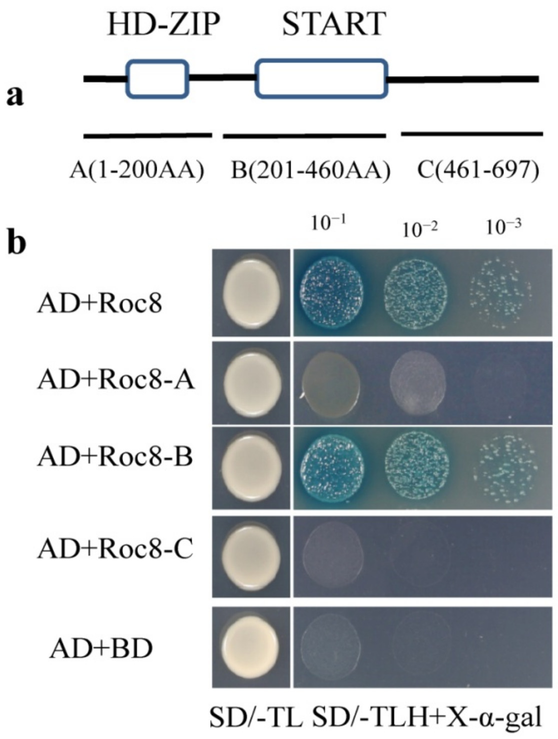

Figure S5. Protein structure, transactivation analysis of Roc8.

a. Top: Roc8 protein structure. Black boxes indicate exons. Bottom: The fusion constructs of Roc8 and its truncated variants. The full ORF of Roc8 was divided into three domains: the N terminus HD-ZIP domain (amino acids 1 to 200, A), the START domain (amino acids 201 to 460, B), and the C terminus (amino acids 461 to 697). The full-length cDNA of Roc8 and DNA fragments responsible for different truncated deletions were introduced into the pGBKT7 vector. BD represents the GAL4 DNA binding domain. Empty pGBKT7 was used as a negative control. The domains of Roc8 are indicated by the N terminus HD-ZIP domain, START domain, and C terminus.

b. Yeast cultures were diluted (1:10 successive dilution series) and spotted onto SD (Leu/Trp) medium and SD (Leu/Trp/His+X-α-gal) medium.

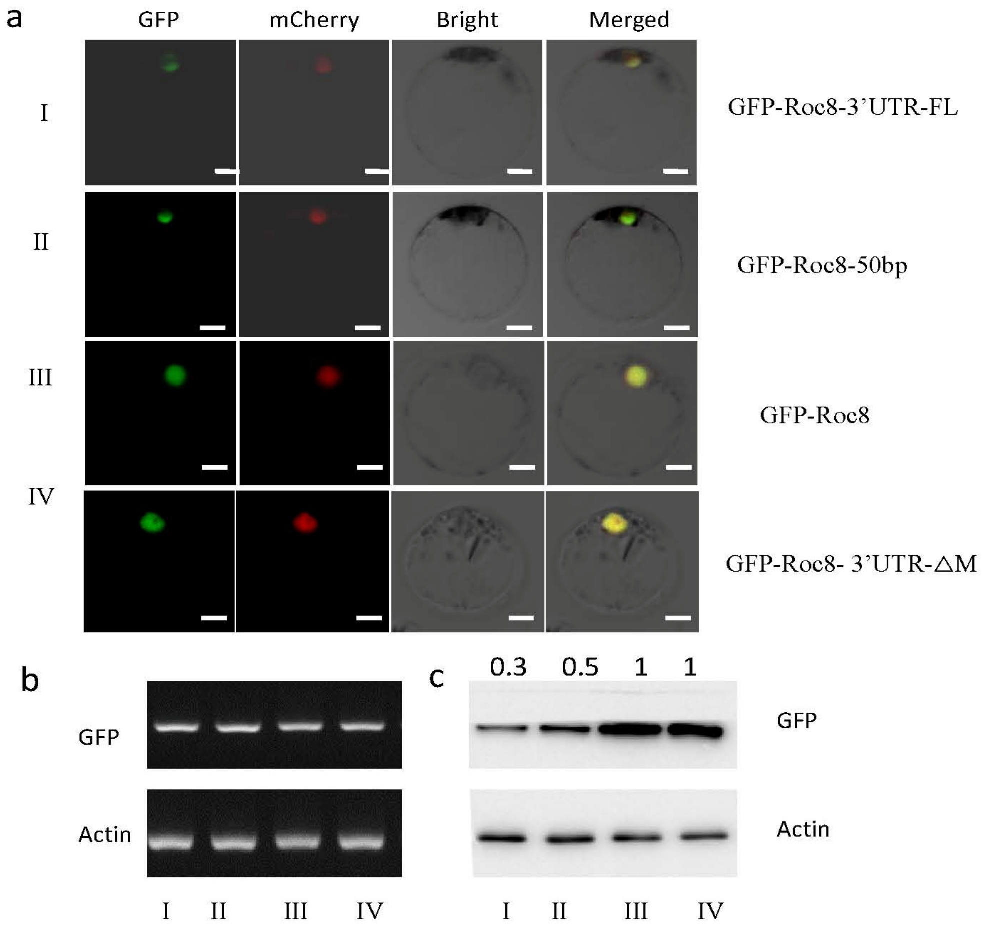

Figure S6. Fluorescence detection of GFP-Roc8 3'-UTR with different fusion constructs using a rice protoplast transient transformation system.

a: To detect the *Roc8* 3'-UTR effect, four vectors containing GFP-Roc8, GFP-Roc8-3'-UTR-FL, GFP- Roc8-3'-UTR-△m, and GFP- Roc8-50 bp were fused into *PAN580* (GFP). The constructs were transformed into rice protoplasts and incubated in the dark at 28°C for 16 h before examination by confocal laser scanning microscopy (Chen et al., 2006). Transient transformation assays using rice protoplasts were conducted similarly for subcellular localization. Images of GFP-Roc8 control and GFP-Roc8 fusion vectors were taken using the same parameters to allow comparison.

b: The protoplast RNAs were also extracted and used to detect GFP expression. Actin was selected as a control. Actin was used as a loading control.

c: The protoplast proteins were also extracted and used to detect the GFP protein amounts. Actin was selected as a control. Actin was used as a loading control, and band intensities were quantified using ImageJ analysis of Western blots. The fold-change shown above the blot is relative to GFP-Roc8 controls normalized by actin in each lane.

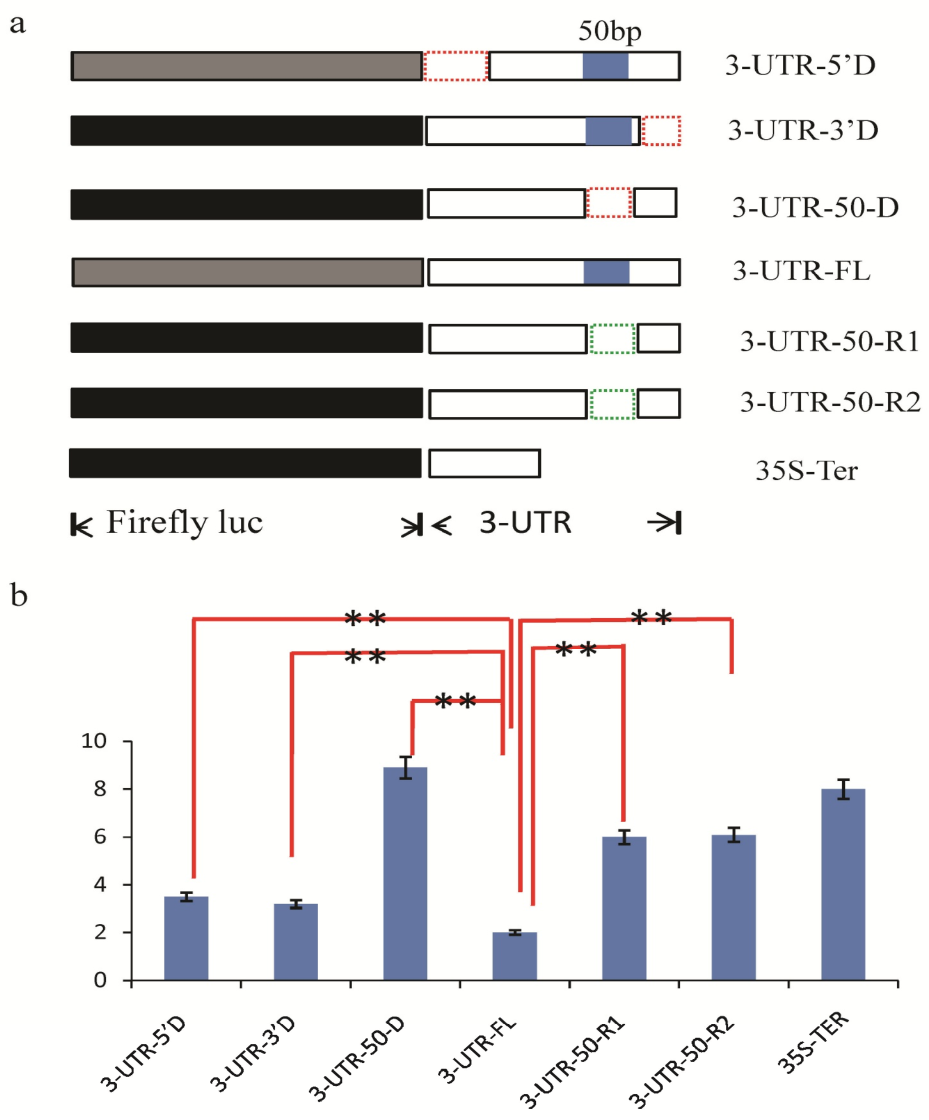

Figure S7. Roc8 3'-UTR regulates luciferase activity.

a: Firefly luciferase reporter constructs fusing the full-length Roc8 3'-UTR deletion variants (3-UTR-5'D, 3-UTR-3'D, 3-UTR-50-D), substitute variants (3-UTR-50-R1 and 3-UTR-50-R2), and the cauliflower mosaic virus 35S terminator (35S Ter, control). The luciferase genes are in dark gray; the 3'-UTRs are in white; the deleted regions are shown with red dotted lines; the substitution sequences are in green gray with diagonals; and the gray rectangle in the 3'-UTR indicates 50 bp.

b: Relative luminescence units versus the transcript levels quantified by qPCR (3-UTR-FL was normalized to 1). The data indicate the relative luminescence/mRNA level calculated from three independent experiments (n=3). **P<0.01 (Student's t-test).

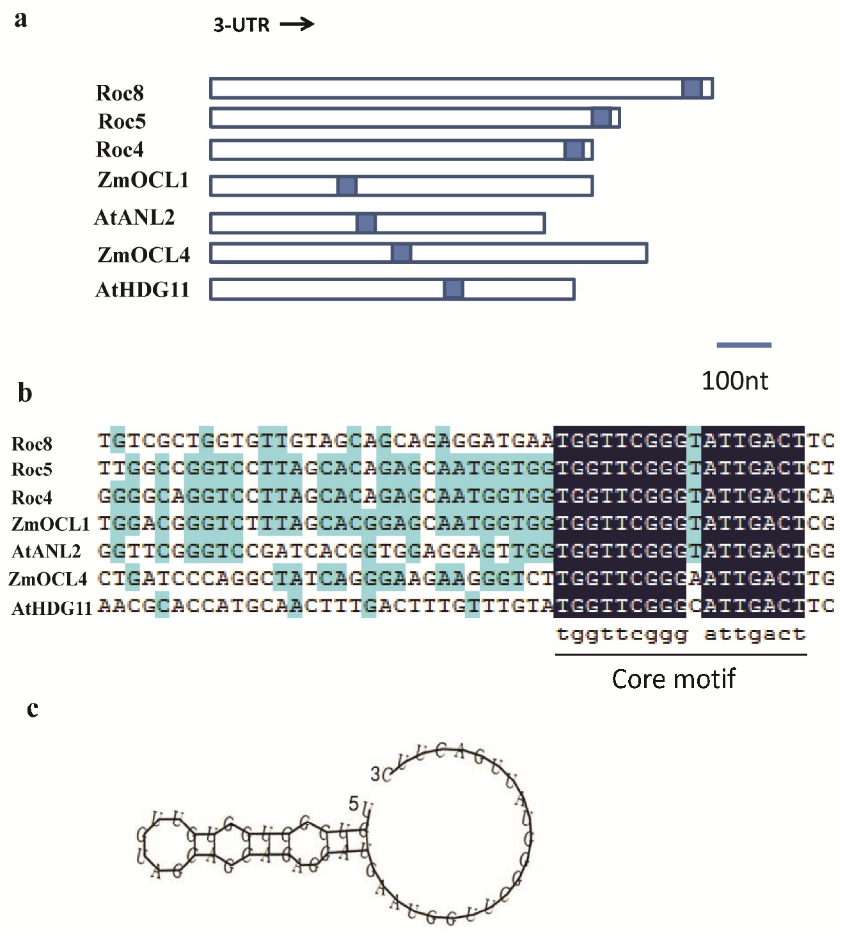

Figure S8. Roc8 3'-UTR sequence analysis.

a: Class HD-ZIP IV 3-UTR core motif analysis (Arabidopsis, Rice, Maize).

b:Class HD-ZIP IV 3'-UTR 50 base core motif alignment. Conserved amino acids are highlighted in black.

c: The 50 bp sequence analysis was conducted by using the secondary structure prediction software RNAfold. The results showed that a long stem-loop structure markedly existed. △G= -6.70 kcal/mol.

http: //rna.tbi.univie.ac.at/cgi-bin/RNAWebSuite/RNAfold.cgi.

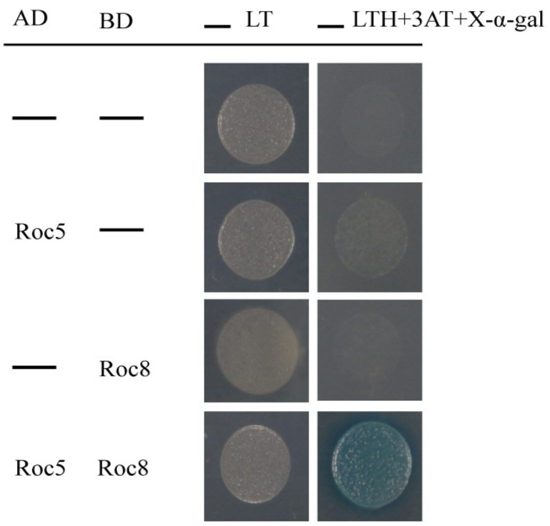

Figure S9. A yeast two-hybrid interaction assay was performed with Roc5 and Roc8.

pGADT7 vector (AD) and pGBKT7 (BD) were used as negative controls. Roc8-DNA binding protein was generated in the pGBKT7 vector (Roc8-BD). A Roc5-activation domain fusion was generated in the pGADT7 vector (Roc5-AD). –TL: SD (Leu/Trp) medium; –LTH+3-AT+X-α-gal: SD (Leu/Trp/His+5 mM 3-AT) medium.

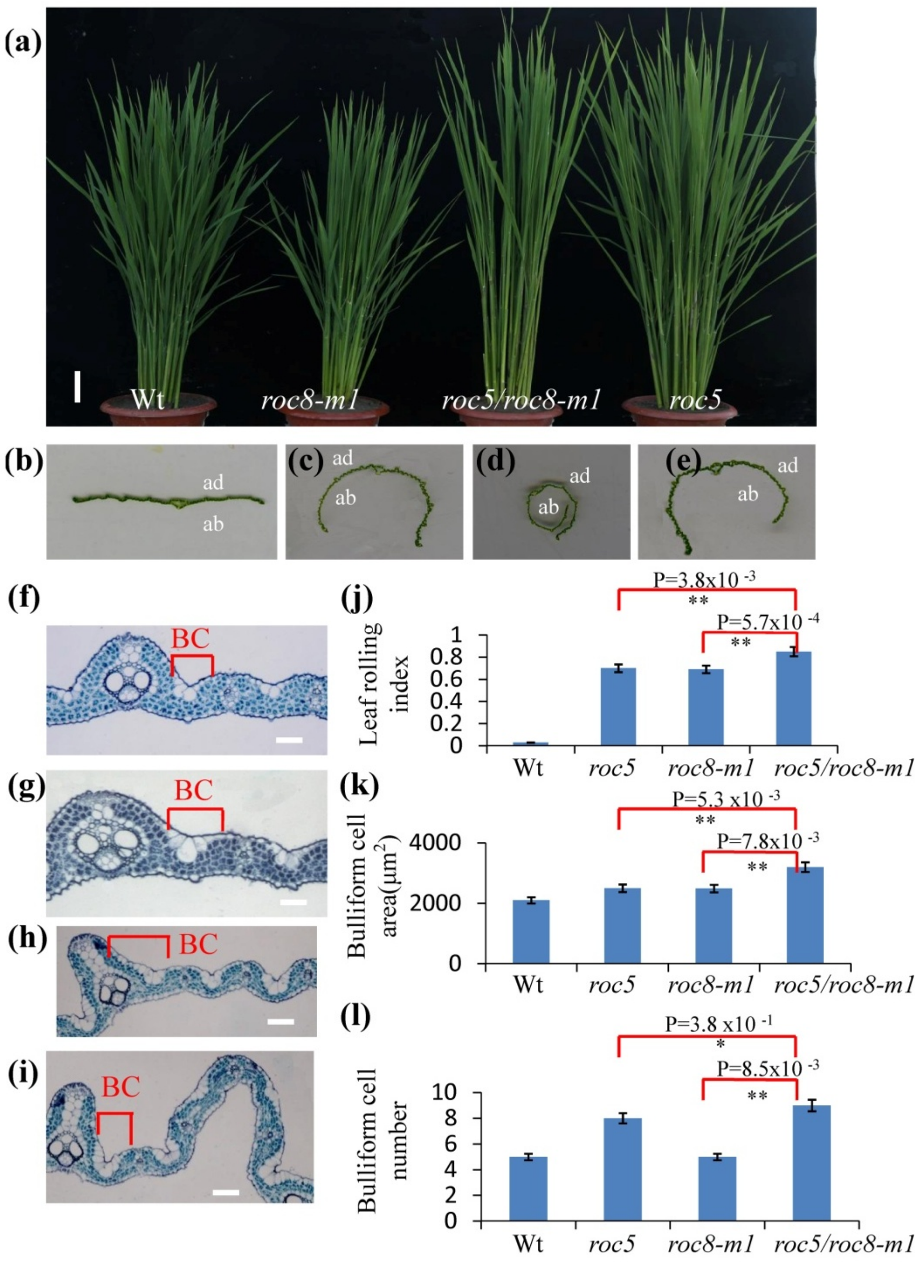

Figure S10. Double mutant analysis of loss-of-function alleles of roc5/roc8.

(a) The plant stature phenotype of Wt, *roc5*, *roc8* and the double mutant (*roc5/roc8*) at the tillering stage.

(b-e) Leaf phenotypes of (b) Wt, (c) *roc8-m1*, (d) *roc5/roc8* double mutant, (e) *roc5* mutant at maturity (ab: abaxial side, ad: adaxial side. bar=5 cm).

(f-i) Representative transverse leaf sections of (f) Wt, (g) *roc8-m1*,(h) *roc5* and (i) *roc5/roc8* double mutants showing the size and number of bulliform cells between a major and minor vein(bar=20 μm).

(j-l) Bulliform cell characteristics of Wt and mutant lines (j, n=10; k: n=10; l, n=10). Bars represent the SD of measurements. Student's t-test was performed to determine significance:**represents $P < 0.01$.

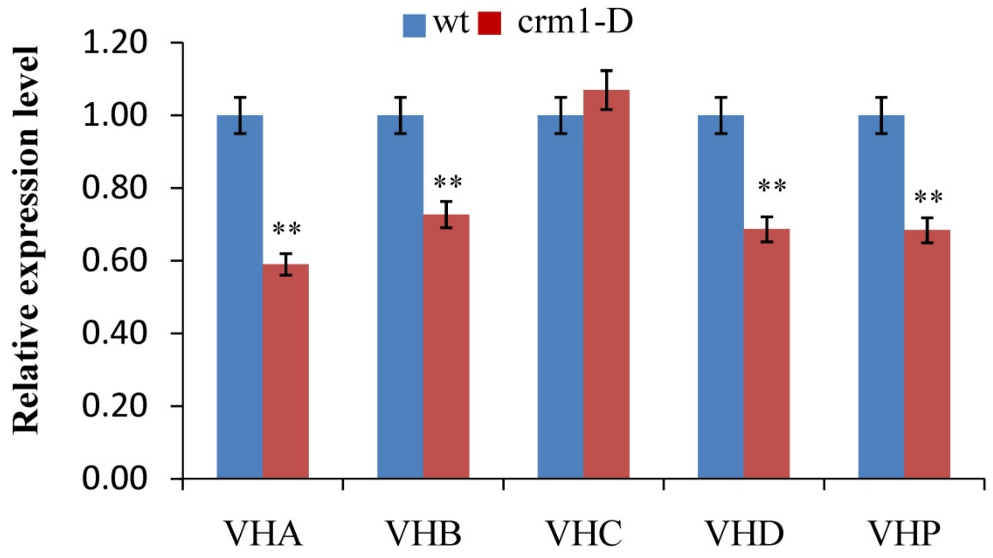

Figure S11. Expression analysis of vacuolar development-related genes.

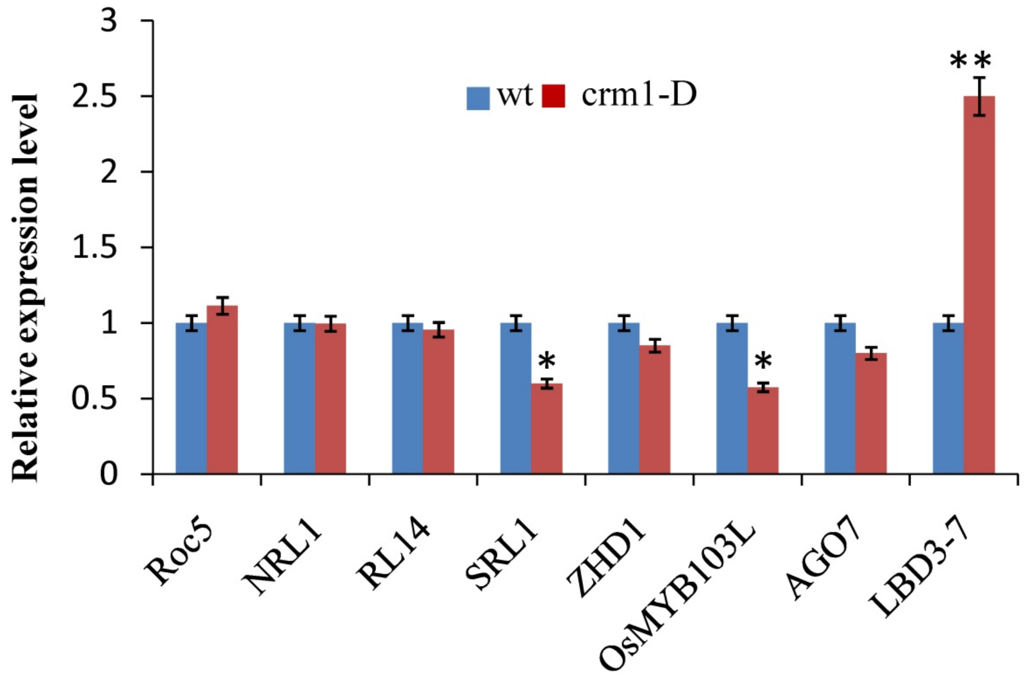

Figure S12. Bulliform cell development-related gene expression analysis in rice.

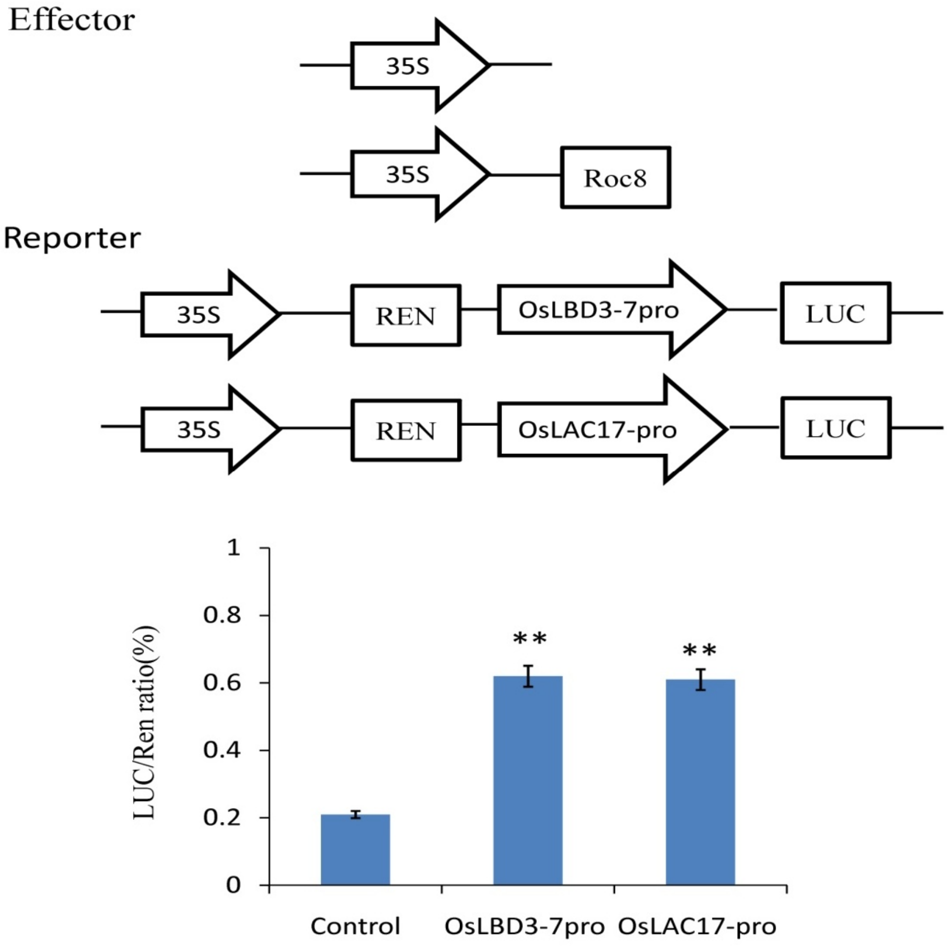

Figure S13. LUC transient transactivation assay in rice protoplasts. Roc8 can significantly activate the transcription levels of OsLBD3-7 and OsLAC17. Data are shown as the means ± SD (n=3). Two star letters represent statistically significant differences at $p<0.01$.

a

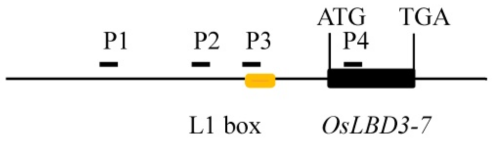

b

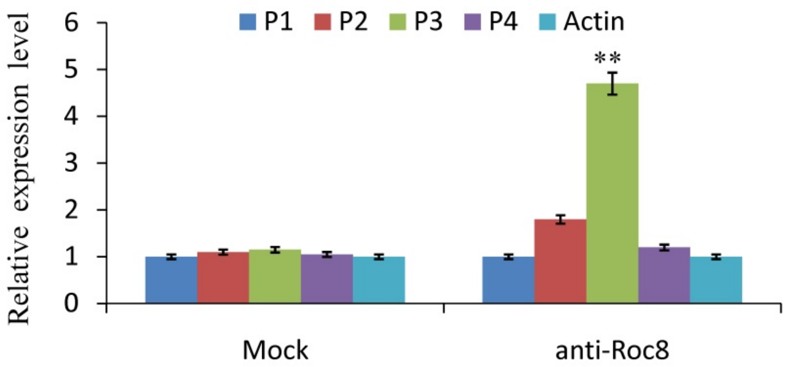

Figure S14. Chip experiment for Roc8 binding to the promoter of the *OsLBD3-7* gene.

a: Schematic of the *OsLBD3-7* promoter region showing regions of the promoter used for PCR amplification. The L1 box is located approximately -679—-617 base pairs upstream of the *OsLBD3-7* start codon.

b: Anti-Roc8 ChIP assays were performed on etiolated seedlings at the 3rd leaf stage. The results showed that Roc8 could bind the P3 region of the *OsLBD3-7* gene.

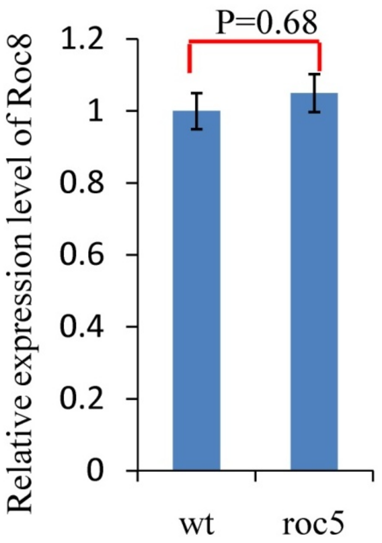

Figure S15. Expression analysis of Roc8 in the r*oc5* mutant and wild type at the 3rd leaf stage.

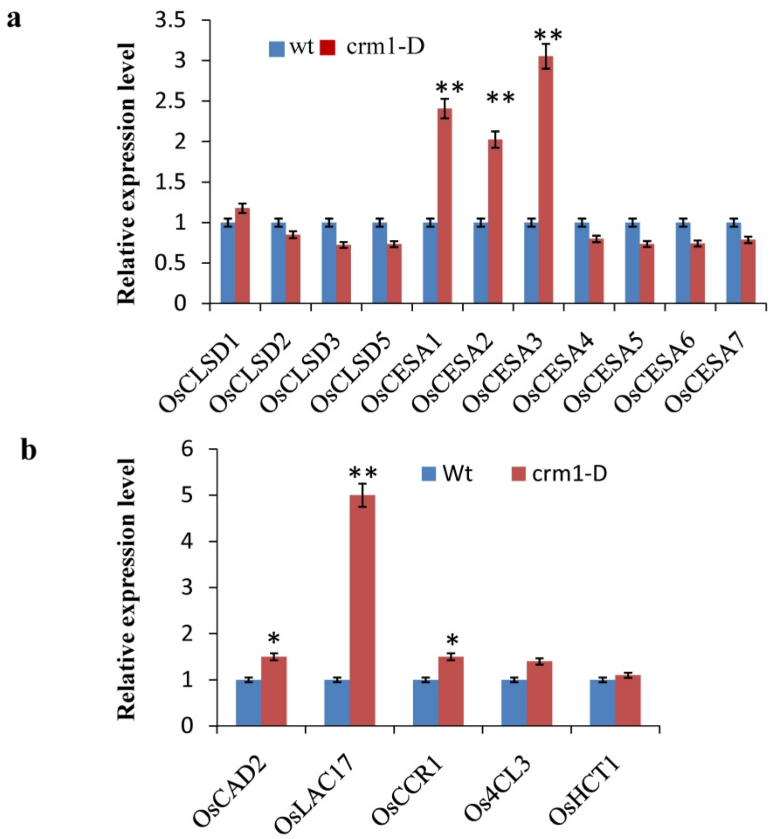

Figure S16. Expression analysis of cellulose-related genes and lignin biosynthesis genes in rice.

a: Expression analysis of cellulose-related gene expression analysis.

b: Expression analysis of lignin biosynthesis-related gene expression analysis.

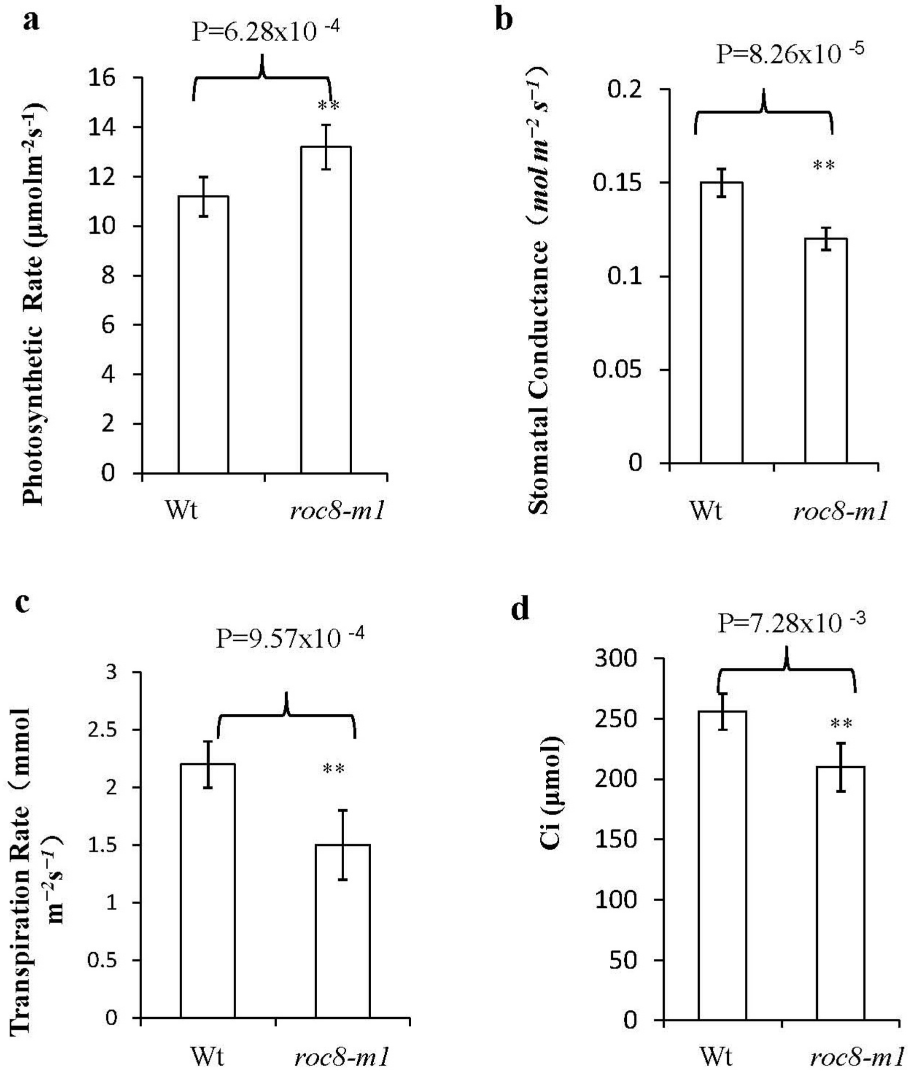

Figure S17. Photosynthesis parameter measurements from the *roc8-m1* plant and wild-type plant at the late tillering stage.

a: Photosynthetic rate comparison. **b:** Stomatal conductance comparison. **c:** Transpiration rate comparison. **d:** Ci comparison. Bars represent the SD of three measurements. Student's t-test was performed using the raw data; two asterisks indicate statistically significant differences at P<0.01.

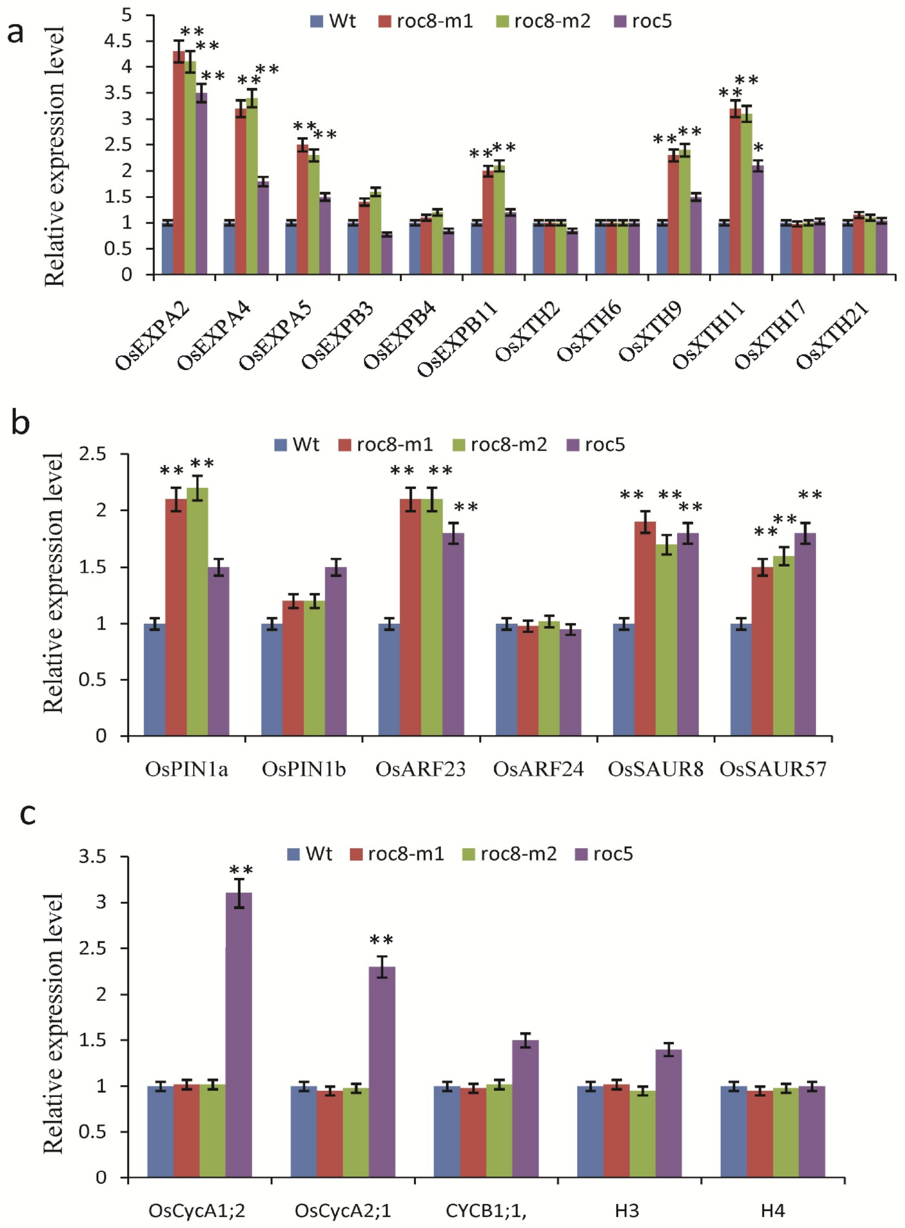

Figure S18. RT-qPCR expression analysis of cell expansion and cell division genes.

a: RT-qPCR analysis of the expression of expansion-related genes and XTH-related genes among Wt and *roc8-m1,roc8-m2,and roc5* plants.

b: RT-qPCR analysis of the auxin-related genes among Wt and *roc8-m1,roc8-m2,roc5* plants.

c: RT-qPCR analysis of the cell division-related genes among Wt and *roc8-m1,roc8-m2,roc5* plants.

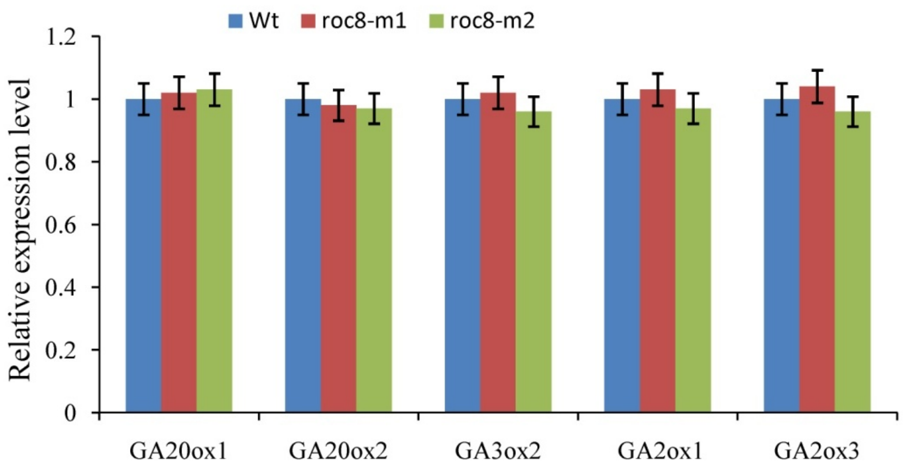

Figure S19. RT-qPCR expression analysis of the gibberellin biosynthesis pathway genes. The results showed that there were no differences in Gibberellin biosynthesis-related genes among wild type (Wt), *roc8-m1 and roc8-m2*. The actin gene was selected as a control (n=3) .

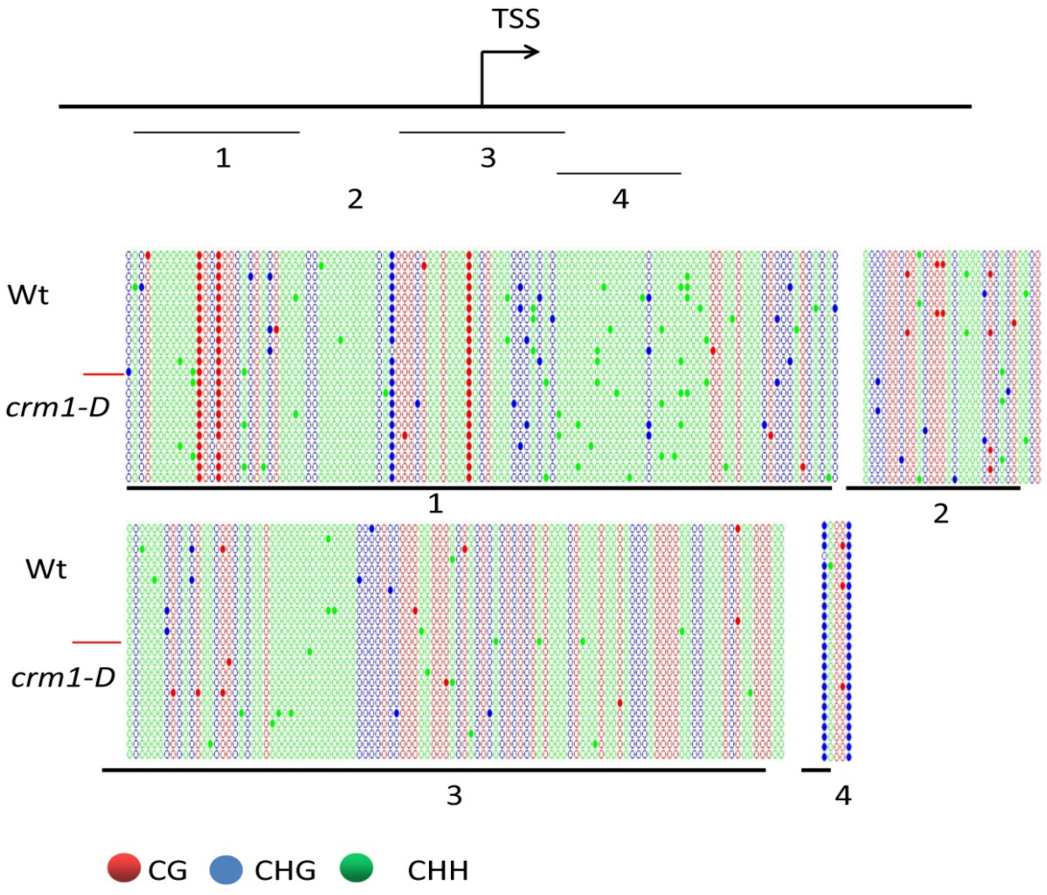

Figure S20. Distribution of three cytosine contexts and methylation patterns in the~800-bp promoter region of Roc8 between wild type and *crm1*-D.

Filled circles, methylated cytosine; empty circles, unmethylated cytosine. There was no obvious methylation difference for three methylation patterns: CHG,CHH and CG.

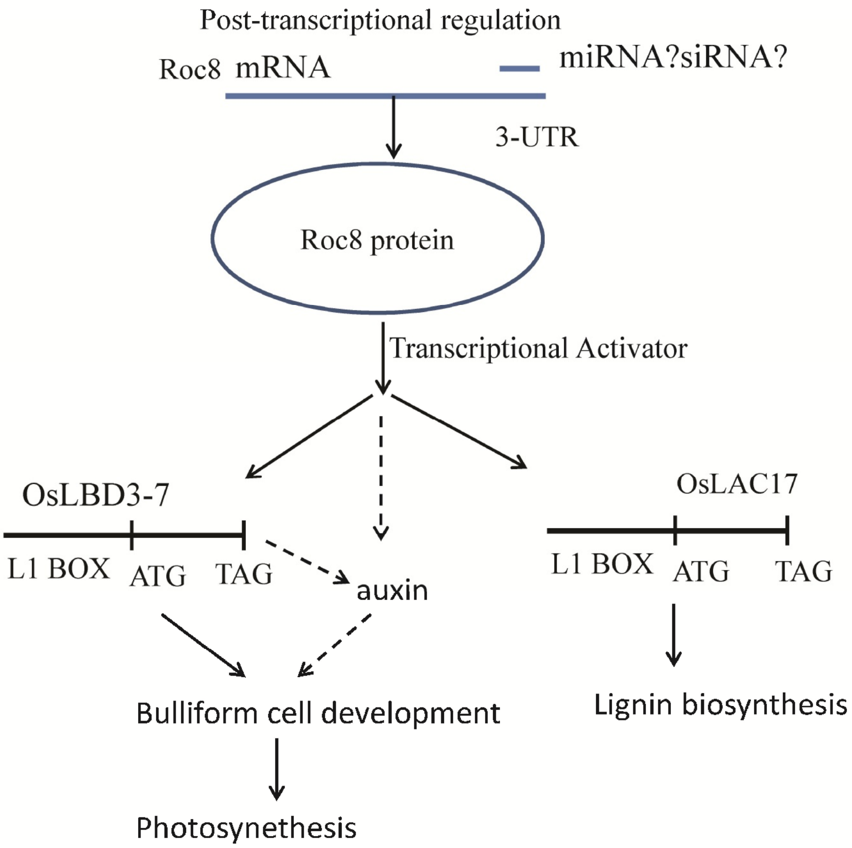

Figure S21. Schematic of the hypothesis illustrating the possible mechanism underlying Roc8 in this study. There were two levels to regulate bulliform cell numbers and lignin contents: transcription and posttranscriptional regulation. The Roc8 3'-UTR is a translational repressor regulator. Roc8 protein itself serves as a transcriptional activator element. Roc8 protein binds to the L1 box region of OsLBD3-7 and OsLAC17(one of the lignin biosynthetic factors). Roc8 perhaps negatively regulated bulliform cell development and influenced plant photosynthesis directly or indirectly dependent on auxin. Additionally, Roc8 positively regulated lignin biosynthesis.

Table S1. Agronomic trait comparison between wild type , *crm1-D* and *roc8-m1* mutants at the mature stage. At least each line with 10 plants was counted.

**Agronomic trait**	**Wild type**	***roc8-m1***	***roc8-m2***	***crm1-D***
Plant height (cm)	115.33±1.21	114.24±1.51	116.45±2.36	112.35±3.14
Panicle length (cm)	23.19±1.75	23.14±1.43	24.25±1.69	23.28±1.28
No. of effective tillers per plant	39.25±3.19	36.33±2.59	37.28±1.68	35.56±1.69
Seed setting percentage (%)	90.01±2.56	92.03±1.23	91.28±3.27	90.89±1.53
1000-grain weight (g)	24.67±0.21	26.14±0.76	27.25±0.28	24.58±0.58
Length of flag leaf(cm)	37.83±1.55	37.84±0.87	36.86±0.48	35.87±0.24
Width of flag leaf(cm)	1.54±0.46	1.48±0.20	1.55±0.18	1.52±0.27
Heading date(days)	175±2	178±3	176±4	179±4
Leaf rolling index	0.02±0	0.62±0.03	0.65±0.04	0.95±0.03

Table S2. Differentially expressed proteins associated with celluose, lignin, epidermis development in leaves

RAP Locus	Ratio(crm1-D/Wt)	P	Protein description	Regulation	L1 box
Proteins invovled in lignin formation/phenylpropanoids metabolism					
OS02G0627100	2.390148076	7.01E-07	Phenylalanine ammonia-lyase,PAL2	Up	NO
OS04G0518100	1.736063839	1.61E-11	Phenylalanine ammonia-lyase,PAL1	Up	NO
OS06G0241400	12.63803271	4.35E-05	O-methyltransferase 1,AAMT1	Up	NO
OS08G0290700	4.812797162	5.28E-25	Trans-resveratrol di-O-methyltransferase,ROMT	Up	NO
OS06G0240800	2.134439044	1.54E-06	Caffeic acid O-methyltransferase,OMT3	Up	NO
OS12G0441600	5.157957908	4.50E-05	O-methyltransferase,ZRP4	Up	NO
OS12G0441300	3.820337246	2.90E-08	O-methyltransferase,ZRP4 like	Up	NO
OS11G0242600	1.648118527	8.89E-06	lucuronoxylan 4-O-methyltransferase 2,GXM2	Up	NO
OS08G0157500	1.527433955	2.79E-06	O-methyltransferase 1,ROMT-9	Up	NO
OS02G0177600	2.017963388	1.18E-07	4-coumarate--CoA ligase 3,4CL3	Up	NO
OS01G0850700	3.52885463	1.86E-05	LAC7	Up	NO
OS09G0262000	4.27E+00	1.25E-05	Cinnamoyl-CoA reductase 1(CCR1)	Up	NO
OS09G0419200	1.744991489	3.40E-10	Cinnamoyl-CoA reductase 1 lkie	Up	NO
OS08G0441500	1.608170087	2.00E-08	Cinnamoyl-CoA reductase 1	Up	NO
OS01G0842400	4.001533979	8.00E-26	LAC17	Up	-679--671
OS11G0708100	2.936504808	5.09E-13	LAC22	Up	-1178--1170
OS09G0400400	0.166824143	8.33E-15	alcohol dehydrogenase,CAD8D	Down	NO
OS09G0399800	0.252035699	3.18E-06	alcohol dehydrogenase,CAD8A	Down	NO

Proteins invovled in cell wall cellulose formation					
OS07G0552800	6.360763736	5.74E-05	CSLF2	Up	/
OS01G0750300	1.854551074	7.32E-15	Cellulose synthase A catalytic subunit 4	Up	/
OS06G0696600	2.033267456	1.72E-06	Xyloglucan endotransglucosylase/hydrolase,XTH22	Up	/
OS08G0237000	1.709388119	4.29E-07	endotransglycosylase/hydrolase,XTH8	Up	/
OS06G0696500	0.426317446	4.70E-07	Xyloglucan endotransglucosylase/hydrolase,XTH2	Down	/
Proteins invovled in cell wall related processses					
OS05G0163300	2.316103942	4.79E-06	Fasciclin arabinogalactan protein 11	Up	/
OS05G0563550	2.149200383	2.02E-10	Fasciclin arabinogalactan protein 13	Up	/
OS01G0159200	1.979436793	1.01E-07	Fasciclin arabinogalactan protein 11 like	Up	/
OS05G0563600	1.500184561	9.37E-07	Fasciclin arabinogalactan protein 13 like	Up	/
Proteins invovled vacuolar development proteins					
OS06G0178900	1.4158221	9.21E-05	Pyrophosphate-energized vacuolar membrane proton pump	Up	NO
OS01G0337500	0.377565801	1.12E-05	Pyrophosphate-energized vacuolar membrane proton pump	Down	NO
OS01G0559600	0.47168643	4.78E-06	Vacuolar-processing enzyme	Down	NO
OS07G0680000	0.537233063	5.98E-06	Vacuolar-sorting receptor 1	Down	NO
OS02G0644000	0.63470583	3.69E-06	Vacuolar-processing enzyme beta-isozyme 1	Down	NO
OS03G0142900	0.645227574	1.73E-05	Vacuolar sorting-associated protein 62	Down	NO

Table S3. Primers used for fine mapping, qRT-PCR, vector construction and etc.

Primers for Fine mapping and complementary test		
Marker	Forward sequence (5’-3’)	Reverse sequence (5’-3’)
Indel6-3	TGTTTATTTTTAGGCCATATGG	GCAAATTTTGTTGCATGGTG
Indel6-5	GTTGGCCTGTTGGGAAGAT	CCGCATCGTCTAATCCCTAA
1-4	GAAATGGTCATCCAATTTC	TAGTATAGAA TATACCAAG
1-6	CTGGAACGCGACGGTGACG	GCCCTAATTAACCCTCTCCA
1-7	TCATCATTTCCATGGTGAAAAA	CTAGTTTGTGCGAACGAGGAC
1-9	CTAACAATAAATCAATGATGC	GGCGGTGTTGATTGAGCTA
Primers for sequencing Roc8 gene		
Roc8-se1-F/R	AAAGATTGGCACGAACAGTCC	AACAAAGCAAAGCAAACCGC
Roc8-se2-F/R	CAGGATGTTCAAGGAGTGCC	GCAGTAGCGGACGAAGTTCA
Roc8-se3-F/R	CCCAAGCATCGTGTCCAAAG	TGTTCTGGCTCGCATTCAAG
Roc8-se4-F/R	ATCAATGGCGGAGAGAGTGG	TGATCTGCTCGACGGTGGTGGT
Primers for Roc8 3'-UTR insertion test		
P1/P2	TCACCCATTGGATTTGTTCC	ACGCATCATCCAGCTTTACC
Primers for qRT PCR for mapping region genes		
Qrt-580F/R	GGGTGGTCCTGTTGAAAAGA	TGCCATGCAACTTGATCTTC
Qrt-590 F/R	TGGGCAGGGATAATGACAAC	CATCATCATCCATCCATA
Qrt-600 F/R(for Roc8)	GTCGTGATGAGCGGTGAG	CTGCTGACGAGGATCTA
Qrt-610 F/R	GCGAGATTCATGGACAAGGT	GCCTCCATGAACGACTT
Qrt-620 F/R	TGCCTTCCACTCCTGTAGGT	TGCCCTCAACACATTAA
Primers for Luciferase assay to detect Roc8 3’-UTR function using rice protoplast tranient transformation		

Fluc fl F/R	actagtATGGAAGACGCCAAAAACATAAAGAAAGG	ggtaccTTACACGGCGATCTTTCCGCCCT
Rluc fl F/R	actagtATGACTTCGAAAGTTTATGATCCAGAACA	ggtaccTTATTGTTCATTTTTGAGAACTCGCTCA
3-UTR-FL(amplification Wt cDNA)	ggacaagtttgtacaaaaaagcaggctaaTCGCCGCCGCCGCCGCCA	ggggaccactttgtacaagaaagctgggtaCCAGCACTCTCTGTTTGT
3-UTR-5'-D (amplification Wt cDNA)	ggacaagtttgtacaaaaaagcaggctaaCTCGCATTCATGGACTCC	ggggaccactttgtacaagaaagctgggtaCCAGCACTCTCTGTTTGT
3-UTR-3'-D (amplification Wt cDNA)	ggacaagtttgtacaaaaaagcaggctaaTCGCCGCCGCCGCCGCCA	ggggaccactttgtacaagaaagctgggtaGTTCATTGAAGGCTTTAC
3-UTR-50-D (amplification crm1-D cDNA)	ggacaagtttgtacaaaaaagcaggctaaTCGCCGCCGCCGCCGCCA	ggggaccactttgtacaagaaagctgggtaCCAGCACTCTCTGTTTGT
3-UTR-50-R1	GGTGGCCATCTGAGCCCTGAGG	TTTACCAAAATGCCAGCAGGCGTAGT
3-UTR-50-R2	TTAATTAGCAACATTAACTCCGATGCTCTGT	TGTCACAACCGGAGTTTTCCCCTTT
35S Ter F/R	ggacaagtttgtacaaaaaagcaggctaaCGCTGAAATCACCAGTCTCTCT	ggggaccactttgtacaagaaagctgggtaCGGTGTGAGGGAACTAGTTTTGAT
Primers for GFP-Roc8 transient transformation detection		

ROC8-F/R(construct GFP-Roc8)	GACGAGCTGTACAGATCTATGGATTTCGGCG ACGAACC	GGGCGGCCGCTTTAAGATCTTCAGGGGTG GTGGCCATG
3-UTR-F/R(amplification wild type or *crm1*-D mutant for construct the GFP-Roc8-3’UTR-FL or GFP-Roc8 -3’UTR-∆ M）	GCGGCCGCCCGGCTGCAGCCACCGAGCTCG CCATTG	CAAATGTTTGAACTGCAGTCCATTTATAAC CGAAATGA
3-UTR-50F/R(amplification 50bp using wild type as template)	GCGGCCGCCCGGCTGCAGTGTCGCTGGTGTT GTA	CAAATGTTTGAACTGCAGGAAGTCAATAC CCGAA
In situ hybridization		
Roc8 sense probe F/R	GGATCCTAATACGACTCACTATAG GGCTCAACGTCGACACCTACGA	CCATTGGCCATGTCAGCGAT
Roc8 anti-sense probe F/R	CTCAACGTCGACACCTACGA	GGATCCTAATACGACTCACTATAGGCCATTGGCCATGTCAGCGAT
For Roc8 subcellular localization		
Roc8 -GFP-F/R	CCGGAGCTAGCTCTAGAATGGATT TCGGCGACGAACC	ACCATGGATCCCCCGGGGGGGTGGTGGCCATGGGCGGAGC
Chip primers		
OsLBD3-7 P1 F/R	TAAGCAGGTCTGCGGCTTAT	CAGGGAAAAACGAGAGCTTG
OsLBD3-7P2 F/R	AAACAAGAAGCCAGGATTGC	TGCACTGGTTTTCCAAAGTG
OsLBD3-7P3 F/R	GCTAGTACTCCCTCCATCCTAAAA	AAAGTACTCCCTCCCTCTTTCAC
OsLBD3-7P4 F/R	CGTGCGTTGGTTTACGTCTT	CATATGTGAAACGACGGGATA
OsLAC17 P1 F/R	AGGGACGAGCATATCAGGAA	TCAATCACCATCAGCAACCT

OsLAC17 P2 F/R	CATGTGGAACCGTTCGTTTT	GACGTTCTCGAGTGCCAAAT
OsLAC17 P3 F/R	AACGATGTGTTTGTATTATATTTATGC	ATGCAAGAATCGACCTTTTGA
OsLAC17 P4 F/R	CTGCGTATCTCCTCGCCTAC	AGCCTAAGCTTTGAGCCACA
OsLAC17 P5 F/R	GCACAACATCTCGTTGCACT	CCGTGAAGTTGTACACGTAGC
Yeast one-Hybrid Analysis		
Gene	Forward Sequence(5’-3’)	Reverse sequence
Roc8-AD F/R	GGAGGCCAGTGAATTCATGGATTTCGGCGACGAACC	CGAGCTCGATGGATCCGATCAGGGGTGGTGGCCAT
Yeast two-Hybrid Analysis		
ROC5-AD-F/R	GGAGGCCAGTGAATTCATGAGCTTTGGGGGCCTC	CGAGCTCGATGGATCCTCAGGCGTCGCACTGCA
Different vectors for transcriptional activity		
Roc8-BD F/R	CATGGAGGCCGAATTCATGGATTTCGGCGACGAACC	GCAGGTCGACGGATCCTCAGGGGTGGTGGCCATGG
Roc8-BD-A F/R	CATGGAGGCCGAATTCATGGATTTCGGCGACGAACC	GCAGGTCGACGGATCCCTCCATGTCGGACACGGGC
Roc8-BD-B F/R	CATGGAGGCCGAATTCCGGCCCATGATGGCCGAGATG	GCAGGTCGACGGATCCCGCGACGAGGGAGGCGTAGC
Roc8-BD-C F/R	CATGGAGGCCGAATTCCTCGGCGTCCCGCACCACATCG	GCAGGTCGACGGATCCTCAGGGGTGGTGGCCATGG
**Primers for luciferase transient transcriptional activity assay**		
OsLBD3-7 F/R	CCCGGGGTTAAACCGCCCACCAACTA	TCTAGAGGGGAGATGGAGATTTGGTT

OsLAC17 F/R	CTGCAGATTTGGCACTCGAGAACGTC	GGATCCAGCCTAAGCTTTGAGCCACA
Primers for Roc8 methylation pattern		
MP1 F/R	AGTAYAGGAGYATGGAGAGGAGAGG	CCTCTTCTRCTACTACTARTTTTCT
MP2 F/R	AGAAAAYTAGTAGTAGYAGAAGAGG	TTCTTRATCTCTRCRTCCCCTCTC
MP3 F/R	TTTGGATTGGATGAGTTGAGGTG	CACCATTTCATCCCTCCATCCAT
MP4 F/R	ATGGATGGAGGGATGAAATGGTG	CAAACARCAAACCCCACACCAT
Primers for Rolling genes expression analysis		
Roc5 F/R	CGCAAGAGGAAGAAGCGATAC	GCTCCAGTTGCGTCTTCATC
NRL1/NAL7 F/R	TCAGTAGTGTAGTGGTGTCGAGTTCA	GCACTCCTTCATGTGAGCTTCA
RL14 F/R	CTCTTTCAGGCATTCCATTGATG	CAACACCTTGTCAGCTTTCAAGC
SRL1 F/R	TCTCCTGCCTCCTCTGTGTG	TAGGAGGGGTGGTGTTGAAG
ZHD1 F/R	TTCCGCACCAAGTTCACCCA	GACGCCGACCTCGTCACAGA
OsMYB103L F/R	CTTAGAAGATGGCCAAACAGCC	TGGCCTCCAAGTTGGATGAT

AGO7 F/R	GCTCAATGTGGAAGCACAGG	ATGTCAGGTCTCCGTGCTATTG
OsLBD3-7 F/R	GCCAGAATGCTCCAGCAAC	GTACACCGGGTCCTGCAC
Primers for Cellulose related genes		
OsCLSD1 F/R	CACAGTCTGTTTGCTTCATGGAG	TAGGAATGCCATTCAAATGACAA
OsCLSD2 F/R	ATGAGCAGTTCTGGCTAATCG	TGATGTCCACTTGACGATGTAGA
OsCLSD3 F/R	TCACCTCCATCTTCCTCCTCG	CATCACCTTCAGCAACCCCTC
OsCLSD5 F/R	TCTTCTCGGGGCAGTTCATC	GGACCACTTCACCTCCAGCA
OsCESA1 F/R	CACTTATCGCCTATTGTGTGC	AAGAGATGGGCAGATGTGC
OsCESA2 F/R	GGTATCCTTGAGATGAGGTGG	GCCTTTGAGGTGACAGTGAA
OsCESA3 F/R	AAGTTCTTCGGTGGGCTCT	TTTCCAGGATGCCAGTAGC

OsCESA4 F/R	TTCCAAAGTCTTCTGTCTGTTCATGGT	CAAGGTCTTCACGTCGAGAGTCT
OsCESA5 F/R	GGACATCCATTCCACTATTGG	GCCTACACCACTCCATCTCAT
OsCESA6 F/R	TTCTTCACATCTCTTCGCTG	CCTTCCAACCAAACCCTT
OsCESA7 F/R	CTCCGTCGAGATCTTCATGAGC	CGCCAAATTGTTAAGCGTGG
Primers for vacuolar related genes primers		
vacuolar H^+-pyrophosphatase (VHP) F/R	TTCCCTCCGTTTCCTATTTGT	CCATCATCGTATCATCCAACC
vacuolar H^+-ATPase subunit A(VHA) F/R	CCGCAGAGGGCAAAGAAGT	TCGGTAGCAGCAAATGGAATG
vacuolar H^+-ATPase subunit B ((VHB) F/R	AGCAGAGATGCTACCCACTGA	ACAGAAGTTGCCGTGACAAAG
vacuolar H^+-ATPase subunit C((VHC) F/R	CGGGGAGACCTATCAGAAGAA	TTCAATACGCAGACCACACTC

vacuolar H^+-ATPase subunit D((VHD) F/R	CAGGGTTACAAGAAGAGGGAGA	GCATCAATACTACGCACCGATT
OsCAD2 F/R	CATCCGTGCAAGGCCAATGTG	GTGCTTCAGTGGGCTGTACAC
Os4CL3 F/R	ATCCTGAGGCGACCAAGAACAC	CGGCAAGATCATCCTTCATCGAG
OsHCT1 F/R	ACCGACGGCATCTCCTCCTTC	CGGTCGATGAACGGCATGACG
OsCCR1 F/R	GCCACTGCAAAGTGTGAGGATGAT	AAATCAGAGGCAGGTGACCCTTCT
OsLAC17 F/R	GCTCTTCCTAGCCCTCTACTCTTG	CATGCTCTTGGTGTTGCACAG
Primers for Gibberellin (GA) biosynthesis related genes		
GA20ox1-F /R	5′-GCCACTACAGGGCCGACAT-3′	5′-TGGTTGCAGGTGACGATGAT-3′
GA20ox2-F/R	5-′CCAATTTTGGACCCTACCGC-3′	5′-GAGAGAAGCCCAACCCAACC-3′

GA3ox2-F/R	5′-TCCTCCTTCTTCTCCAAGCTCAT-3′	5′-GAAACTCCTCCATCACGTCACA-3′
GA2ox1-F/R	5′-TGACGATGATGACAGCGACAA-3′	5′-CCATAGGCATCGTCTGCAATT-3′
GA2ox3-F/R	5′-TGGTGGCCAACAGCCTAAAG-3′	5′-TGGTGCAATCCTCTGTGCTAAC-3′
Primers for expansion, auxin related genes and cell cycle protein elated genes		
OSEXPA2	GGGCACTCCTACTTCAACCT	TAGGAGTTGCTCTGCCAGTT
OsEXPA4	GGGCACTCCTACTTCAACCT	CTGGAAGGAGAGGCTCTGG
OsEXPA5	CGCATCGCTCTCTTCAAGG	TTGAAGTAGGAGTGCCCGTT
OsEXPB3	TTCTCGTCGATGACCTCCTG	AGGGTGGTTGACGCATCTTA
OsEXPB4	ATCAGATGCACCAAGGACCA	GCCACCGGGTAGTAGTTCAT
OsEXPB11	CCAGGTCCAGTACAGAAGGG	CGAAGTAGAACGGGTTTGCC

OsXTH2	TCAGCATGCACATCAAGCTC	GTTCGTCTGCAGGATGTACG
OsXTH6	GCGTTCAAGTCCAAGACCTC	CGTGGGTGTTCCAATCCTTC
OsXTH9	TGGAGGTCCAAGAACACGTA	AACTCCAGGTCCACCTCATC
OsXTH11	ACCTTCTACTTGTCGTCGCA	TGCTGTGGGTTCCAGATGAT
OsXTH17	TACGAGAAGTTCGACGTGGT	CCGAACAGGTAGGTGTCCTT
OsXTH21	CCTACACCCTCCAGACCAAC	CCTCTCGTACCTCCTGATCG
OsPIN1a	GGACCATCACCCTCTTCTCC	CAGATGATGCACTGGAGCAC
OsPIN1b	CATCACGCTCTTCTCCCTCT	GAGCGTGTACCAGATGATGC
OsARF23	TGCTGTTAACACAGGGACCA	CTGGAGCCTCTTCACCTTCA
OsARF24	TGCCAAGTTTGGTGGGAATG	TTGGCTCAATATGCCCAAGC

OsSAUR8	GCCAACCTGAAGCAGATCCT	GAAGCAGCAGCAGGAAGTC
OsSAUR57	CCTACAGGAACCTCCGATCC	TTGTTGCTGATCACGTAGCG
OsCycA1;2	CGAGACGATGTCGATGTGTG	ATACGCAGGTTCTCGTTTGC
OsCycA2;1	ACCACCGCATAGTCAAATCGTT	ACCACAATGTCACCACCTCGTGA
CYCB1;1,	GCTTCAGTGGAATCTCACCG	GTGGTGCTTGAGAGTGTCAG
H3	GCAAGTACCAGAAGAGCACG	GTTGGTGTCCTCGAAGAGAC
H4	GCAAGTACCAGAAGAGCACG	AGAGGTTGGTGTCCTCGAAG

Methods S1.Transmission Electron Microscopy (TEM) Analysis.

Leaf samples of wild-type and *crm1*-D were harvested at the mature stage. Leaf sections were fixed in primary fixation solution (4% glutaraldehyde). M phosphate buffer (pH 7.2), samples were post-fixedwith 1% osmium tetroxide. After rinsing with phosphate buffer, the samples were dehydrated in a graded ethanol series before transitions and infiltrations were processed. The polymerization reaction was carried out overnight at 70℃.The sections were sliced to 50 nm with an ultramicrotome (MT-X, RMC, USA), stained with 2% uranyl acetate and Reynolds' lead citrate, and observed under transmission electron microscopy (JEM-1010, JEOL, Japan).

Methods S2. Histology and cytology observation.

Samples including wild type, *crm1-D*, Roc8-overexpression lines(OEs), *roc5* knock-out lines, *roc8* knock-down lines, and a double mutant between *roc8* and *roc5* knock-out lines in 10th leaves were collected for paraffin section analysis. The samples were fixed, washed and then dehydrated as previously described (Zou et al., 2011). Sections (approximately 5–10 μm thick) were cut with a microtome (Leica RM2155), stained with 1% (w/v) safranin O (Amresco) and 1% (w/v) fast green FCF (Merck), examined with a fluorescence microscope (Zeiss AXIO Imager A1), and photographed. The areas and numbers of bulliform cells were counted using AxioVision 4.6 software (Carl Zeiss AG) (n=10).

To observe the arrangement of bulliform cells in rice leaves, the middle part of leaves was selected at the 10th leaf stage, and the 1-2 cm adaxial surface was treated. First, silica and water were added, ground gently, and then soaked in 95% (V/V) ethanol for approximately 1 day, and the solution was changed every 4 hours to remove chlorophyll. Then, the samples were washed with water, soaked in 1 N NaOH for 12 h to extract cell contents, and fixed with Carnoy's solution for 12 h. The samples were then stained overnight in 1% (W/V) toluidine blue O (Hernandez et al., 1999). After washing with water, the dirty was removed and left for microscopic examination (Zeiss Axio imager A1) and photographing.

Methods S3. *In situ* hybridization.

Transverse and longitudinal sections through the shoot apex of six-day-old seedlings were obtained according to an *in situ* hybridization procedure (Roche, Cat#SP6/T7). The procedure was described as seven parts: fixation and embedding of tissue, preparation of RNA probes (*Roc8* anti-sense or sense probe), preparation for *in situ* hybridization, washes, detection, and imaging.

Methods S4. RNA preparation and quantitative real-time (qRT)-PCR analysis.

The leaves were isolated using an RNA extraction kit according to the manufacturer's instructions (TianGen Cat#DP432). First-strand cDNA was reverse transcribed using an oligo(dT) primer. The transcription of a series of related genes was assessed using qRT-PCR with the rice actin gene as the reference sequence. Each 20-μL reaction contained 2 μL of the reverse-transcribed product, 0.2 μM each primer, and 1× SYBR green PCR master mix (Takara Co. Ltd., Otsu, Japan). The reaction conditions were as follows: 95°C/30 s, followed by 40 cycles of 95°C/5 s, 60°C/30 s, and 72°C/30 s.

Changes in transcription were calculated using the $\Delta\Delta^{CT}$ method (Livak and Schmittgen,2001).

Methods S5. Protein extraction, sodium dodecyl sulfate-polyacrylamide gel electrophoresis (SDS-PAGE), and Western blotting.

Leaf material was homogenized in lysis buffer (25 mM Tris-HCl, pH 7.6; 0.15 M NaCl; 2% SDS; and 0.01% 2-mercaptoethanol). The sample amounts were standardized by 5 μg fresh weight. The protein samples were separated by 10% SDS-PAGE. After electrophoresis, the proteins were transferred to a PVDF membrane (EMD Millipore Corp., Billerica, MA) and incubated with specific antibodies. The signals were detected using a Pierce ECL Plus Western Blotting Detection Kit (Thermo Fisher Scientific, Waltham, MA) and visualized with an imaging system (ChemiDocTMXRS; Bio-Rad, Hercules, CA). Roc8 antibodies were synthesized by BGI (Shenzhen, China).

Methods S6. Transcriptome Data Analysis.

Wild-type and *crm1-D* leaf RNAs at the 3rd leaf stage were extracted by using TRIzol reagent. Each sample was assayed in triplicate. After that, the RNA samples were sent to the Novogene sequencing company for transcriptome analysis.

Methods S7. Subcellular localization of Roc8 in rice.

To investigate the subcellular localization of Roc8, we first amplified the Roc8 ORF without its termination codon by reverse transcription (RT)-PCR using primers Roc8 -GFP-F/R, cut by XbaI and SmaI restriction enzymes, and then inserted it into the PAN580-GFP vector (Zhang et al., 2011), placing Roc8 upstream of the green fluorescence protein (GFP) coding sequence to create an in-frame fusion of Roc8 and GFP (Roc8-PAN580). We expressed a Roc8:GFP fusion construct or its negative control (PAN580-GFP) in young rice leaf protoplasts. The protoplasts were incubated in the dark at 28°C for 16 h before examination by confocal laser scanning microscopy (Zhang et al., 2011).

Methods S8. Yeast Two-Hybrid assay and Yeast One-Hybrid assay.

To detect the transcriptional activation activity of Roc8, the Roc8 coding sequence was divided into three parts (Roc8-A, Roc8-B, Roc8-C) according to the Roc8 domain structure. The Roc8-A, Roc8-B, Roc8-C, and Roc8 full-length coding sequences (CDS) were amplified with the respective primers for cloning via the In-Fusion HD cloning system (Clontech, no.639650). *Roc8* inserts were shuttled into the bait (pGBKT7) vectors for expression in yeast cells (AH109). The transcriptional activation activity of Roc8 in different domains was checked and further confirmed. The interaction between Roc8 full CDS (Roc8) and Roc5-AD vectors was also checked and further confirmed on SD (Leu/Trp) and selective SD (Trp/His/Leu) media in addition to 5 mM 3-AT. A yeast one-hybrid test was conducted as follows. *OsLAC17* promoted an L1 sequence (AACATTTA). The 3XL1 sequence of *OsLAC17* was synthesized and fused with the pHis2 vector (3XL1 vector). Two vectors (Roc8-pGADT7 and 3XL1 vector) were simultaneously transformed into yeast (AH109) and grown into SD medium (Trp/Leu) and SD medium (Trp/Leu/His) supplemented with 5 mM 3-AT.

Methods S9. Rice protoplast transformation assay.

To detect the *Roc8* 3'-UTR effect, four vectors containing GFP-Roc8, GFP-Roc8-3'-UTR-FL, GFP- Roc8-3'-UTR-△m, and GFP- Roc8-50 bp were fused into *PAN580* (GFP). The constructs were transformed into rice protoplasts and incubated in the dark at 28°C for 16 h before examination by confocal laser scanning microscopy. Transient transformation assays using rice protoplasts were conducted similarly for subcellular localization. Images of GFP-Roc8 control and GFP-Roc8 fusion vectors were taken using the same parameters to allow comparison. The protoplast RNAs and proteins were also extracted and used to detect GFP expression and GFP protein levels. Actin was selected as a control.

Methods S10. Luciferase assay to detect Roc8 3'-UTR function using a rice protoplast transient transformation system.

The reporter in p2CGW7 was replaced with firefly or Renilla luciferase to generate p2FLGW7 or p2RLGW7 for the 3'-UTR assay with the Fluc fl and Rluc fl primers. The Roc8-3'-UTR and 3'-UTR deletion versions were constructed using the primers listed in Table S3. The 35S terminator, a negative control, was amplified from pJAM1849 with the 35S Ter forward and reverse primers. To replace 50 bp with other DNA fragments, two unrelated sequences were amplified with the 3-UTR-50-R1 forward and reverse primers and with the 3-UTR-50-R2 forward and reverse primers from rice genomic DNA. The fragments used for transient expression were cloned into pDONR221 using the Gateway BP clonase II enzyme (Invitrogen) and were subsequently moved into p2FLGW7 with the Gateway LR clonase II enzyme (Invitrogen). The 35S Ter was cloned into p2RLGW7 for normalization (Shen et al., 2017).

Methods S11. Bisulfite sequencing analysis.

To determine the DNA methylation levels in the *crm1*-D plants and the wild types, genomic DNA was isolated from leaves at 60 days after germination using the DNeasy Plant Mini Kit (Qiagen). Bisulfite treatment was performed with the EpiTect Bisulfite kit (Qiagen) following the manufacturer's instructions. The treated DNA was used for PCR amplification with primers at the Roc8 locus. The primers were designed by Methyl Primer Express software (Applied Biosystems). Primers for bisulfite sequencing are listed in Table S3. All PCR fragments were cloned into the pGEM-T easy vector (Promega), and 20 clones for each fragment were sequenced. The sequencing data were analyzed using web-based kismeth bisulfite analysis software.

Methods S12. Lignin content measurement.

Leaf lignin contents were measured according to the method of acidolysis titration with sulfuric acid (Xiong et al., 2005). The main steps were as follows: (1) Sample extraction: Firstly, smashed the samples, took 0.1-0.2g into the centrifuge tube, added 10ml of 1% acetic acid solution, shook well and centrifuged; washed the precipitate with 5ml of 1% acetic acid, then added 3-4ml of ethanol and ether to mix (volume ratio 1:1), soaked for 3min, discarded the supernatant and washed for 3 times.

Secondly, evaporated the precipitate in the centrifuge tube in the boiling water bath, added 3ml of sulfuric acid with a mass fraction of 72% to the precipitate, mixed well with a glass rod, standing at room temperature for 16h, dissolved all cellulose, then added 10ml of distilled water to the tube, mixed well with a glass rod, placed in the boiling water bath for 5min, added 5ml of distilled water and 0.5ml of barium chloride solution with a mass fraction of 10% after cooling, shook well and centrifuged. Finally, the precipitate was washed twice with distilled water, 10 ml of sulfuric acid with a mass fraction of 10% and 10 ml of 0.1 mol/l potassium dichromate solution were added to the washed lignin precipitate, and the tube was placed into a boiling water bath for 15 min, mixed at any time, and cooled to standby. (2) Sample determination: First, all the substances in the cooled tube were transferred to the beaker for titration, and the residual part was washed with 15-20 ml distilled water. Then, 5 ml of KI solution with a mass fraction of 20% and 1 ml of starch solution with a mass fraction of 0.5% were added to the beaker and titrated with 0.2 mol/l sodium thiosulfate. Notice: Ten milliliters of sulfuric acid with a mass fraction of 10% and 10 ml of 0.1 mol/l potassium dichromate solution were added as blank samples by titration alone. According to the above methods, the lignin contents of the three lines of *crm1-D*, *roc8-m1*, and wild type were measured.

Methods S13. Luciferase transient transcriptional activity assay in rice protoplasts.

The luciferase activity assay was used to detect whether Roc8 can activate OsLBD3-7 and OsLAC17. The promoters of OsLBD3-7 and OsLAC17 were amplified and fused into the PGreen II-0800-LUC vector as a reporter. The full cDNA of Roc8 was inserted into the empty vector PAN580. PAN580 was used as a negative control. Luciferase®Reporter Assay System (Promega, Madison, U.S.A) was used to detect the luciferase activity according to the instructions using a rice protoplast transformation assay. The ratio between LUC (firefly luciferase) and Ren (Renilla luciferase) was recorded as described (Zhang et al., 2020). Transient transformation assays using rice protoplasts were conducted similarly for subcellular localization.

Methods S14. Chromatin immunoprecipitation (ChIP) and quantitative PCR analysis.

Chromatin immunoprecipitation was conducted as described (Clontech, Cat#640166). In brief, rice seedlings (2 g) of *Nipponbare* as a control were fixed with 1% formaldehyde in phosphate-buffered saline (PBS) at room temperature for 10 min with gentle agitation. After two washes with 40 mL ice-cold PBS, the samples were ground to extract the proteins and DNA. The chromatin solution was then sonicated to shear the DNA into fragments. After centrifugation, the chromatin pellet was resuspended in 300 mL buffer containing 50 mM Tris-HCl (pH 8.0), 10 mM EDTA, 1% SDS, 1 mM PMSF (phenylmethanesulfonyl fluoride) and protease inhibitors. The above solution was divided into three portions, and then 900 mL buffer [1.1% Triton X-100, 1.2 mM EDTA, 16.7 mM Tris-HCl (pH 8.0) and 167 mM NaCl] was added. After adding 40 mL of salmon sperm-sheared DNA to each chromatin sample with

gentle agitation and overnight incubation at 4°C, the pellet was rinsed three times and resuspended in a buffer containing 50 mM Na3PO4 (pH 8.0), 167 mM NaCl, 10 mM imidazole and protease inhibitors. The input solution was then used for immunoprecipitation using Roc8 antibodies. The DNA fragments were cleaned using a PCR DNA purification kit (Qiagen). Real-time quantitative PCR was employed to quantify the immunoprecipitated genomic DNA. Quantitative measurements of various regions of *OsLBD3-7* and *OsLAC17* were performed using SYBR Green PCR master mix (Applied Biosystems). Three repetitions were conducted.

Methods S15. *Roc8* 3'- UTR deletion construction.

To detect the *Roc8* 3'-UTR effect, a series of *Roc8* 3'-UTR-related vectors were constructed. The Roc8-FL-∆U vector was constructed by ligation the Roc8 full-length coding sequence without the 3'-UTR (amplification using wild type as a template) and the pCambia2300-modified vector (plus the rice actin promoter) from our lab' construct. The Roc8 full-length coding sequence containing the 3'-UTR was amplified using wild type as a template and ligated with the pCambia2300-modified vector (Roc8-FL). The full-length *Roc8* gene, including its 3'-UTR (deletion 50 bp), was amplified using the *crm1-D* mutant as a template and ligated with the pCambia2300-modified vector (Roc8-FL-∆m). The construction vectors were transformed into *Nipponbare* calli, and regenerated plants were obtained. Quantitative PCR assays of Roc8 mRNA levels and Roc8 protein in T_1 transgenic and corresponding near-isogenic null segregants were conducted (n=3).

Methods S16. Field evaluation for agronomic traits.

Wild-type, *crm1-D*, and *roc8* knockdown lines were grown in the field. Agronomic traits such as plant height, seed setting rate, 1000-grain weight, panicle length, heading date, tiller numbers, length of flag leaf, width of flag leaf, and leaf rolling index were investigated.

Plant height (cm) isequal to the height from the top of thepanicle to the ground at the mature stage. The seed setting rate(%) is defined as the ratio of the number of full grains to the total number of grains (including empty grains).The 1000-grain weight (g) is defined as the weight of 1000 grains. The tiller numbers were counted after the tiller stage.

The length or width of flag leaves were counted after flowering.

The leaf rolling index (LRI) was measured on the 20th day after flowering. These measurements are as follows:

Expand the leaf blade and determine the greatest width of the leaf blade (Lw).At the same site, measure the natural distance of the leaf blade margins (Ln).

LRIs are calculated as follows: LRI (%) = (Lw − Ln)/Lw × 100.At least 10 plants from each line were counted.

Methods S17. Photosynthetic parameters measurement.

The photosynthetic rate, stomatal conductance, transpiration rate, and Ci of the wild-type, *crm1-D*, and Roc8 knockdown lines were measured at the heading stage using a photosynthetic LiCOR-6400 instrument with 500 μmol s^{-1} flow velocity, 28°C

leaf chamber, and 2000 μmol s^{-1} light quantum flux density. At least three replicates were carried out for each line.

References

Hernandez, M.L., Passas, H.J., and Smith, L.G. (1999) Clonal analysis of epidermal patterning during maize leaf development. *Developmental Biology*. 216(2), 646-658.

Livak, K.J. and Schmittgen, T.D. (2001) Analysis of relative gene expression data using real - time quantitative PCR and the 2(- Delta Delta C(T)) method. Methods, 25, 402–408.

Shen, J., Liu, J., Xie, K., Xing, F., Xiong, F., Xiao, J., Li, X., and Xiong, L. (2017) Translational repression by a miniature inverted-repeat transposable element in the 3' untranslated region. *Nature Communications*. 8, 14651.

Xiong, S.M., Zuo, X.F. and Zhu, Y.Y. (2005) The determination of cellulose, hemi - cellulose and lignin in rice hull. Detect. Anal. 8, 40–41.

Zhang, K., Su, J., Xu, M., Zhou, Z., Zhu, X., Ma, X., Hou, J.et al . (2020) A common wild rice - derived BOC1 allele reduces callus browning in indica rice transformation. *Nature Communications*. 11, 443.

Zhang, Y., Su, J., Duan, S., Ao, Y., Dai, J., Liu, J., Wang, P.et al . (2011) A highly efficient rice green tissue protoplast system for transient gene expression and studying light/chloroplast - related processes. *Plant Methods* , 7, 30.

Zou, L.P., Sun, X.H., Zhang, Z.G., Liu, P., Wu, J.X., Tian, C.J., Qiu, J.L., and Lu, T.G. (2011) Leaf rolling controlled by the homeodomain leucine zipper class IV gene Roc5 in rice. *Plant Physiology*. 156(3), 1589-1602.
